# Mechanochemically Engineered Bimetallic PtNi/CeO_2_ Catalysts for Enhanced Methane Steam Reforming

**DOI:** 10.1021/acscatal.5c06508

**Published:** 2026-02-04

**Authors:** Andrea Braga, Marina Armengol-Profitós, Laia Pascua-Solé, Lluís Soler, Isabel Serrano, Ignacio J. Villar-Garcia, Virginia Pérez-Dieste, Enrico Tusini, Andrea De Giacinto, Anna Zimina, Jan-Dierk Grunwaldt, Jordi Llorca, Núria J. Divins

**Affiliations:** † Department of Chemical Engineering, Institute of Energy Technologies, and Center for Research in Multiscale Science and Engineering, EEBE, 16767 Universitat Politècnica de Catalunya, Eduard Maristany 10-14, Barcelona 08019, Spain; ‡ ALBA Synchrotron Light Source, Carrer de la Llum 2-26, Cerdanyola Del Vallès, Barcelona 08290, Spain; § Institute for Chemical Technology and Polymer Chemistry, 150232 Karlsruhe Institute of Technology (KIT), Engesserstraße 20, Karlsruhe 76131, Germany; ∥ Institute of Catalysis Research and Technology, 150232 Karlsruhe Institute of Technology (KIT), Hermann-von-Helmholtz-Platz 1, Eggenstein-Leopoldshafen 76344, Germany

**Keywords:** bimetallic nanoparticles, ceria, ball milling, methane steam reforming, hydrogen, NiPt

## Abstract

Bimetallic PtNi/CeO_2_ catalysts were successfully synthesized via a mechanochemical approach, specifically ball milling, and evaluated for methane steam reforming (MSR). A fractional factorial design of experiments was employed to systematically explore the effects of key milling parameters—milling frequency, milling time, and ball-to-powder ratio—on the catalysts’ structural properties and catalytic performance. The catalysts were characterized by X-ray diffraction, H_2_ temperature-programmed reduction, transmission electron microscopy, and Raman spectroscopy. Catalytic activity tests were performed in a plug flow reactor under a high gas hourly space velocity (200,000 mL g_cat_^–1^ h^–1^) at a steam-to-carbon ratio of 2 between 700 and 950 °C. The mechanochemically synthesized catalysts were benchmarked against those prepared via incipient wetness impregnation. The most active milled catalysts achieved a methane conversion rate of ca. 22 mol CH_4_ g_Ni_^–1^ h^–1^ at 700 °C (83.5% methane conversion for a PtNi/CeO_2_ mechanochemically synthesized), outperforming the impregnated counterpart (64% methane conversion under the same reaction conditions). Notably, increasing the milling intensity resulted in enhanced catalytic activity, with milling frequency emerging as the most influential factor—correlating with the formation of smaller NiO particles. To elucidate the role of Pt addition, *in situ* X-ray absorption near-edge structure (XANES) and near-ambient pressure X-ray photoelectron spectroscopy (NAP-XPS) measurements were conducted on the most active milled catalysts under MSR conditions. NAP-XPS revealed surface segregation of Pt during MSR, alongside an inhibitory effect on solid carbon deposition, suggesting the potential for a coke-resistant catalyst. These findings highlight the power of mechanochemical synthesis in tuning catalyst properties, offering a scalable and efficient route to high-performance catalysts for methane reforming and hydrogen production.

## Graphical abstract

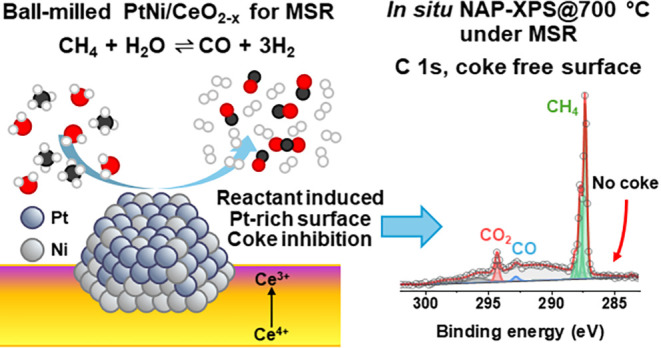

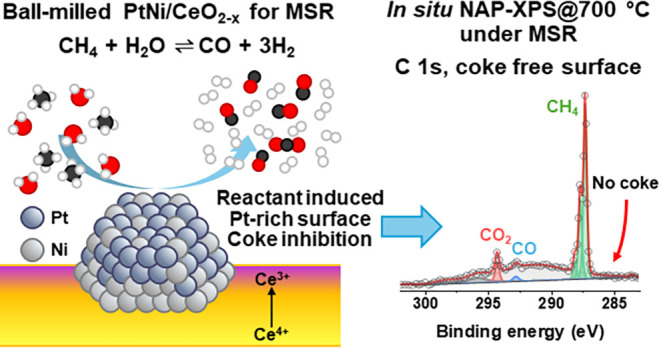

## Introduction

The transition toward a more sustainable future requires several changes in energy production. In this regard, methane is an important molecule as it may be converted into hydrogen and synthesis gas (syngas, a mixture of CO and H_2_), which are both employed in the production of higher-value compounds and energy-related applications.^1^ Industrially, methane steam reforming (MSR, reaction 1) coupled with the water–gas shift reaction (WGSR, reaction 2) is the principal method to produce both syngas and H_2_ using natural gas as feedstock.^2^ The utilization of biomethane obtained from biogas is one of the possibilities for reducing the emissions of CO_2_ from the steam reforming of methane.^3,4^

1 $${\mathrm{CH}}_{4(g)}{+H}_{2}O_{\left(g\right)}⇄{\mathrm{CO}}_{\left(g\right)}{+\mathrm{3 H}}_{2(g)},\Delta H_{298K}^{0}{=+\mathrm{206 kJ mol}}^{-1}$$

2 $${\mathrm{CO}}_{\left(g\right)}{+H}_{2}O_{\left(g\right)}⇄{\mathrm{CO}}_{2(g)}{+H}_{2(g)},\Delta H_{298K}^{0}{=-\mathrm{41 kJ mol}}^{-1}$$

The most used catalysts for MSR are based on nickel supported on alumina due to the high activity of Ni and low price compared to noble metals.^5^ One of the main problems with these catalysts is the high rate of coke formation on Ni and the formation of a NiAl_2_O_4_ spinel phase, which is inert and difficult to recover once it is formed.^6–8^ Several strategies exist to mitigate the formation of carbon, such as working at high H_2_O/CH_4_ ratios at the expense of higher energy demand, decreasing the Ni particle size,^9,10^ promoting or modifying the support material with different oxides,^11–13^ and adding a second metal to enhance the activity and stability of the Ni catalysts.^14–16^

Among the different possibilities, bimetallic formulations based on Pt–Ni alloys showed enhanced activity and resistance to coke formation compared with monometallic Pt- or Ni-based catalysts. Low Pt loadings enhanced methane reforming of Ni/Al_2_O_3_ and Ni/MgAl_2_O_4_ catalysts mostly by decreasing the Ni particle size, promoting the reducibility of Ni species, keeping Ni reduced,^17^ and decreasing the rate of carbon formation.^18–22^ Some works reported Pt–Ni/Al_2_O_3_ catalysts with similar methane conversions compared to monometallic Ni ones, although their stability and reducibility were improved by Pt addition.^23–25^ Platinum addition to Ni/CeO_2_ catalysts has been studied for the dry reforming of methane and the steam reforming of ethanol. Higher activities were attained due to the higher dispersion of Ni, and increased coke resistance was observed due to the formation of more amorphous carbon with respect to graphitic carbon because of the presence of Pt.^26,27^

The increased stability provided by Pt has also been investigated in several computational studies. In principle, flat bimetallic Pt–Ni surfaces are predicted to show lower activity compared with monometallic Ni surfaces due to an increased energy barrier for the last steps of methane dissociation.^28,29^ This is correlated with the shift of the d-band center to more negative values for the Pt overlayers on top of the Ni substrate, which is related to lower surface reactivity.^30,31^ Nevertheless, the increased energy barrier for the C–H bond splitting is also related to a higher energy barrier for carbon deposition, so that Pt-rich Pt–Ni surfaces show low coke deposition rates compared to pure Ni surfaces.^29,32,33^ The higher activity of bimetallic Pt–Ni catalysts is thus attributed to a structural effect related to the increased dispersion of the Ni active phase resulting from the Pt addition, and to an increased stability for the limited formation of coke.^18^

Regarding the support material, cerium oxide, used as a promoter or as a support, showed very good resistance to coke formation for MSR.^34–36^ The redox properties of CeO_2_ allow for the rapid formation of oxygen vacancies, providing an oxidizing environment for the gasification of carbon species.^37–39^ Furthermore, CeO_2_ interacts strongly with transition metals and actively participates in the reforming reaction. Oxygen vacancies and the metal-ceria interfaces play key roles in H_2_O activation, providing favorable reaction pathways, limiting the production of coke, and increasing the kinetics of CO and CO_2_ formation.^9,40^ The strong interaction between CeO_2_ and the metals can also be exploited to anchor metal clusters and nanoparticles, limiting particle aggregation.^41–44^

The preparation method is of great importance in developing robust catalysts. Industrially, catalysts are prepared by impregnation and precipitation methods, requiring solvents, multistep processing, and high temperatures. New methods have been explored recently for more sustainable catalyst production. In this regard, mechanochemistry is gaining importance as a green chemistry method for preparing heterogeneous catalysts.^45–49^ The advantages of these techniques rely on the solventless approach, room-temperature material processing, simple and cheap instrumentation, fast syntheses and preparations, and single-step procedures for preparing advanced materials. Most importantly, milling processes can be readily scaled up from laboratory to industrial settings.^50,51^

In this article, we have studied bimetallic PtNi/CeO_2_ catalysts prepared by ball milling for syngas production from methane steam reforming. Despite ball milling being a simple technique, the proper investigation of the impact of the different milling parameters requires a large number of experiments when one variable at a time is optimized. Thus, in this work, we have chosen a fractional factorial design of experiment (DoE) to rationalize the choice of the milling parameters and to efficiently define optimal synthesis parameters.^52,53^ On selected samples, we have further carried out *in situ* synchrotron characterization by performing near-ambient pressure X-ray photoelectron spectroscopy (NAP-XPS) and X-ray absorption spectroscopy (XAS) to correlate the structure and the activity of the catalysts.

## Materials and Methods

### Catalysts Preparation

High-surface area cerium oxide (CeO_2_) was prepared with a hydrolysis-hydrothermal synthesis as reported elsewhere.^54^ Briefly, to 100 mL of Ce­(NH_4_)_2_(NO_3_)_6_ (Acros Organics) aqueous solution (1.0 mol L^–1^) under stirring, 195 mL of NH_3_ solution (0.35 mol L^–1^) were added dropwise. The solution was left under reflux for 4 h, and a yellow precipitate formed. The precipitate was separated by centrifugation and mixed in a Teflon vessel in 90 mL of NH_3_ (2.0 mol L^–1^) to carry out a hydrothermal treatment at 180 °C for 2 h in a stainless-steel autoclave. The product was filtered and washed with distilled water, dried at 110 °C for 24 h, and calcined at 950 °C for 5 h in air.

The nominal metal loading of the bimetallic PtNi/CeO_2_ catalysts was 9 wt %, with 0.9 wt % Pt and 8.1 wt % Ni (0.11 weight ratio, 0.033 atomic ratio). The ball-milled (BM) samples were prepared in a FRITSCH Mini-Mill Pulverisette 23 mill, using a single ZrO_2_ ball (Ø = 15 mm, *m* = 10 g) in a 15 mL ZrO_2_ jar, by simultaneously mixing the metal precursors, Pt­(NO_3_)_4_(NH_3_)_2_ (PtNN) (Alfa Aesar) and Ni­(CH_3_COO)_2_ (NiAc_2_) (Sigma-Aldrich), with the CeO_2_ powder in the jar. For samples prepared using long milling times, we stopped the milling process every 15 min to manually mix the powder and allow it to cool down. The milled powders were dried at 120 °C for 12 h and calcined at 400 °C for 4 h in air. Samples are named *n*-PtNi/CeO_2_ (*n* = 1–5; see details in Table 1 for the nomenclature). Additionally, monometallic Pt/CeO_2_ and Ni/CeO_2_ samples were prepared using the milling parameters of the best-performing bimetallic catalyst (see the section Catalytic Activity).

**Table 1:** Fractional Factorial 2^(3–1)^ and One Central Point Design of the Experiment Table for the Synthesis Parameters

Sample name	Frequency (Hz)	Time (min)	BPR	Milling energy
**1-PtNi/CeO**_**2**_ **(−–+)**	15	5	20	∝3.4 × 10^05^
**2-PtNi/CeO**_**2**_ **(−+−)**	15	45	5	∝7.6 × 10^05^
**3-PtNi/CeO**_**2**_ **(+–−)**	50	5	5	∝3.1 × 10^06^
**4-PtNi/CeO**_**2**_ **(000)**	32.5	25	12.5	∝1.1 × 10^07^
**5-PtNi/CeO**_**2**_ **(+++)**	50	45	20	∝1.1 × 10^08^

Impregnated samples were prepared as references with the same metal loadings by incipient wetness impregnation (IWI). The sample PtNi/CeO_2_ seq-IWI was prepared by sequentially impregnating the metal precursors: first, NiAc_2_ with acidified water (with HNO_3_, pH = 4) was impregnated on CeO_2_. The resulting powder was calcined at 400 °C for 4 h, and then the PtNN precursor was impregnated, with a final calcination step at 400 °C for 4 h.^24^ A second IWI catalyst was prepared by the simultaneous impregnation of NiAc_2_ and PtNN on CeO_2_ and is named PtNi/CeO_2_ co-IWI.

### Design of Experiment (DoE)

Three parameters were identified as the most influential in the milling synthesis: the milling frequency (ν), the milling time (*t*), and the ball-to-powder mass ratio (BPR). To efficiently investigate the effect of each parameter in the synthesis of PtNi/CeO_2_–BM catalysts and to rapidly investigate the wide range of milling intensities available, a fractional factorial 2^(3–1)^ experimental design with a central point was used to define the synthesis parameters.^52,53^

The milling frequency was varied from 15 to 50 Hz, the milling time ranged between 5 and 45 min (stopping the milling every 15 min to cool down the jar and mix the powders), and the BPR ranged between 5 and 20 by varying the mass of powder loaded. These values are the lowest and highest values studied for each level, represented as “–” and “+” signs, respectively, and “0” corresponds to the intermediate value. Therefore, in the samples’ names, we use the symbols “+”, “–”, and “0” to note if we used “high”, “low”, or “intermediate” milling parameters, and they are written in the order: “frequency”, “time”, and “BPR”. The milling parameters used for the syntheses were generated by the DoE module in Minitab19 (LLC, 2023) and are listed in Table 1.

The combination of the milling parameters can be correlated with different amounts of energy transferred to the powders during the synthesis. From the interactions and formulas described in refs. 55 and 56, we derived a relationship between the milling parameters frequency (ν), time (*t*), and BPR and the energy delivered, which is shown in eq 3 (details are provided in the Supporting Information, SI). According to this relationship, we calculated an estimation of the milling energy on a mass basis for each sample, as listed in Table 1.

3 $$E\propto \nu^{3}\cdot t\cdot BPR$$

Additionally, monometallic Pt and Ni catalysts were prepared with the same milling parameters as those used for the best bimetallic catalysts, and these samples were named following the same nomenclature. Therefore, 5-Ni/CeO_2_(+++) and 5-Pt/CeO_2_(+++) correspond to monometallic Ni and Pt catalysts prepared at 50 Hz for 45 min and a BPR of 20.

### Characterization

Inductively coupled plasma optical emission spectroscopy (ICP-OES) was used to quantify the effective metal loadings and was carried out with a PerkinElmer Optima 2300 spectrometer. For the digestion procedure, 50 mg of sample was mixed with 2 mL of HNO_3_, 2 mL of H_2_SO_4_, and 500 mg of NH_4_Cl. The digestion was assisted by microwave heating at 240 °C for 30 min.

The structure and phase composition of the samples were measured by powder X-ray diffraction (XRD) using a Bruker D8 Advance equipped with a Cu cathode operating at 40 kV and 40 mA. The diffractograms were collected with 0.01° steps and 4 s per step. The size of NiO and Pt nanoparticles was estimated by fitting the NiO (200) reflection at 43.3° and the Pt (111) reflection at 39.8° with pseudo-Voigt-I functions in OriginPro 9.0 using the Scherrer equation (eq 4) with *K* = 0.9:

4 $$d=\frac{K\cdot \lambda }{FWHM\cdot \cos\left(\theta \right)}$$

The estimation of CeO_2_ size and residual strain was performed using the Williamson–Hall analysis (W–H),^57^ as reported in the SI.

Hydrogen temperature-programmed reduction (H_2_-TPR) was used to examine the reduction properties of PtNi/CeO_2_ catalysts. About 50 mg of the sample was heated to 350 °C in Ar (15 °C min^–1^, 30 mL min^–1^), held for 30 min, and then cooled to 50 °C. The H_2_-TPR was carried out in 10% H_2_/Ar (10 °C min^–1^, 50 mL min^–1^) from 50 to 850 °C using a Chemstar-TPx apparatus fitted with a thermal conductivity detector (TCD).

Raman spectroscopy was used to study the oxygen vacancies and the Pt–NiO interaction induced by the milling process. A Renishaw IN-VIA apparatus with a 532 nm laser operated at 1 mW and a 50× objective was used to measure the Raman signal of the calcined samples, collecting several spectra in different regions to account for inhomogeneities.

The microstructure of selected catalysts was studied using high-resolution transmission electron microscopy (HRTEM) with an FEI TECNAI F20 S/TEM microscope at 200 kV. The samples were dispersed in methanol and drop-cast on holey-carbon copper grids.

*In situ* X-ray absorption spectroscopy (XAS) measurements were conducted at the CAT-ACT beamline at the Karlsruhe Institute of Technology (KIT) Light Source (Karlsruhe, Germany)^58^ in fluorescence mode and using a silicon drift detector (Vortex-90EX) to measure the Ni K-edge (8333 eV). The XAS data evaluation was performed using Athena and Artemis data analysis software using the IFEFFIT package.^59^ The extended X-ray absorption fine structure (EXAFS) data χ­(k), collected at room temperature, were extracted, background subtracted, and subsequently analyzed in the k^2^-space range of 2.5–9 Å^–1^. The Fourier-filtered R-space used for the modeling is 1.0–2.7 Å. The amplitude reduction factor (*S*_0_^2^) estimated from the fitting of a Ni foil is 0.81. The Ni metal structure used to model the Ni–Ni shell was derived from the structure reported in the literature (ICSD collection code 260169). The catalysts were diluted in SiC in a 1:1 ratio, pressed, and sieved (100–200 μm fraction). About 10 mg of the mixture were loaded into quartz capillaries (inner diameter of 1 mm), and a thermocouple was placed inside the capillary. The capillary was mounted on the cell available at the CAT-ACT beamline, where the sample was heated up by resistive heating.^60^ A mass spectrometer was connected to the reactor outlet to monitor the evolution of the products during the reaction. XANES spectra were acquired during: (i) a temperature-programmed reduction carried out in 10% H_2_/He from room temperature (RT) to 700 °C (50 mL min^–1^, 5 °C min^–1^), holding for 30 min at 700 °C. X-ray absorption near-edge spectra (XANES) were acquired during the heating ramps (every 10 °C). After that, the samples were cooled down in He to room temperature. (ii) A light-off experiment from RT to 700 °C (5 °C min^–1^) in a gas mixture composed of about 1300 ppm of CH_4_ and 2800 ppm of H_2_O in He (S/C ≈ 2, 50 mL min^–1^), holding for 1 h at 700 °C and cooling in He. XANES spectra were recorded every 10 °C during the heating ramp, and a linear combination analysis (LCA) was performed on the XANES spectra measured during the H_2_-TPR step to identify the reduction temperature of selected samples. The LCA was performed using the Ni foil and the first spectrum of each TPR as the reference for nickel oxide species, as the catalysts studied are composed of oxidized nanoparticles interacting with the CeO_2_ support. Extended X-ray absorption fine structure (EXAFS) measurements were acquired on the as-prepared samples and at room temperature after the temperature-programmed reduction at 700 °C and after the MSR tests.

Synchrotron near-ambient pressure X-ray photoelectron spectroscopy (NAP-XPS) was performed at the beamline BL-24 CIRCE of the ALBA synchrotron (Barcelona, Spain). The NAP-XPS end station is equipped with a Phoibos NAP150 electron analyzer from SPECS. The samples were pressed on a gold mesh to compensate for charging and mounted on a sample holder with a thermocouple touching the surface. An infrared laser was used to heat the samples. High-resolution spectra were collected at steps of 0.1 eV with an energy pass of 10 eV. During the experiments, the gaseous mixtures were dosed with independent mass flow controllers, keeping a dynamic pressure of 1 mbar with an automatic valve in the analysis chamber connected to a turbopump. A mass spectrometer interfaced with the first pumping stage of the detector was used to monitor the composition of the gaseous atmosphere and analyze the reactants and products. The experiments consisted of three steps: (i) dosing 10 mL min^–1^ of O_2_ from room temperature to 400 °C (10 °C min^–1^) to clean the surface from adventitious carbon; then, the sample was cooled to 100 °C and O_2_ was removed. (ii) An activation step consisting of dosing 10 mL min^–1^ of H_2_ from 100 to 700 °C (10 °C min^–1^), holding for 30 min at 700 °C, and then H_2_ was evacuated. (iii) The methane steam reforming mixture was dosed at 700 °C, flowing 1.25 mL min^–1^ of CH_4_ and 2.5 mL min^–1^ of H_2_O (steam-to-carbon ratio S/C = 2). During each step, a survey spectrum and high-resolution Ce 3d + Ni 2p, Pt 4f + Ni 3p, and the O 1s and C 1s regions were measured. Data from two sampling depths were obtained by using two different X-ray photon energies to scan each spectral region. The photon energies were chosen to generate photoelectrons with kinetic energies of 215 and 450 eV, probing the surface and subsurface regions, respectively. In Table S1, the photon energies used and the corresponding inelastic mean free paths (IMFP) for the electrons emitted from Ni, Pt, and Ce regions with kinetic energies of 215 and 450 eV are reported. Generating photoelectrons with the same kinetic energy allows consideration of the fact that the photoelectrons are generated from the same depths in heterogeneous samples. Energy correction was performed by measuring all the regions at 1335 eV and using the energy shift to set the Ce^4+^ u‴ peak to 916.9 eV.^61^ The Ce 3d region calibrated at 1335 eV was used as a reference for the regions measured at the other energies. Data analysis was performed with CasaXPS 2.3.25 ^62^ using the line shape GL(50), Spline-Shirley, and Shirley backgrounds. The regions scanned were Ce 3d + Ni 2p, the O 1s, and the Pt 4f + Ni 3p. Cerium oxide deconvolution was done with the model described by Mullins et al.^61,63^ using three doublets for Ce^4+^ and two doublets for Ce^3+^ species. Nickel was deconvoluted as a mixture of NiO and Ni­(OH)_2_ following the work of Biesinger et al.^64^ Due to the overlap between the Ce 3d_5/2_ and Ni 2p_1/2_ regions, only Ni 2p_3/2_ peaks were used for quantification purposes. For metallic Ni, the main line shape used was LF­(0.75, 1.57, 80, 50), while for metallic Pt, the line shape used was LF­(1, 2, 10, 30). The Pt doublet was modeled with a doublet with the relative areas and peak splitting constrained using the signal of the monometallic Pt/CeO_2_ catalyst as a reference. The atomic fraction quantification of Ce, Ni, and Pt was done by considering the integrated peak areas, the corresponding relative sensitivity factors (RSF) considering the ionization cross-section for each element and energy as reported in,^65^ and correcting for the X-ray photon flux at each photon energy.

### Catalytic Testing

The catalytic performance of methane steam reforming was studied by mixing 100 mg of catalyst with SiC in a quartz reactor (plug flow reactor, inner diameter of 8 mm) with a bed volume of 1 cm^3^. Before the catalytic testing, the catalysts were activated from room temperature to 700 °C (10 °C min^–1^, holding for 30 min) in 10% H_2_/N_2_ (50 mL min^–1^). The MSR reaction mixture was composed of 112.5 mL min^–1^ of N_2_, 150 mL min^–1^ of steam, and 75 mL min^–1^ of CH_4_ (CH_4_:H_2_O:N_2_ = 1:2:1.5, S/C = 2), giving a total flow of 337.5 mL min^–1^, corresponding to a flow-to-weight ratio of F/W = 202.5 L g_cat_^–1^ h^–1^ and a space velocity of GHSV = 20250 h^–1^. Liquid water was pumped into lines heated at 120 °C, where water was evaporated, using an HPLC pump (Knauer). The effect of temperature on the catalytic activity was studied between 700 and 950 °C at temperature steps of 50 °C, staying one hour at each step. Reaction products were monitored online using a micro gas chromatograph (GC, Agilent Technologies 3000A) equipped with a 5 Å molecular sieve and a PoraPlot U column. Methane conversion (X_CH4_) was calculated using eq 5. Only H_2_, CO, CO_2_, H_2_O, and CH_4_ were detected at the reactor outlet.

5 $$\mathrm{XCH}4=\frac{{ṅ}_{{\mathrm{CH}}_{4}}^{\mathrm{IN}}-{ṅ}_{{\mathrm{CH}}_{4}}^{\mathrm{OUT}}}{{ṅ}_{{\mathrm{CH}}_{4}}^{\mathrm{IN}}}$$

## Results and Discussion

### X-ray Diffraction

X-ray diffraction (XRD) patterns of calcined DoE bimetallic samples are shown in Figure 1a, while XRD patterns of the IWI counterparts and monometallic 5-Ni/CeO_2_(+++) and 5-Pt/CeO_2_(+++) are shown in Figure S1. In general, four phases were identified in the samples: CeO_2_, NiO, metallic Pt, and metallic Ni, which formed during the calcination due to the nickel acetate decomposition.^66,67^ In all samples, peaks corresponding to the CeO_2_ fluorite were identified. Additionally, metallic Pt reflections were identified in the samples with the lowest milling energies: 1-PtNi/CeO_2_(−–+), 2-PtNi/CeO_2_(−+−), and 3-PtNi/CeO_2_(+–−), as larger Pt nanoparticles (NPs) formed, and their sizes ranged from 35 to 20 nm. In sample 4-PtNi/CeO_2_(000), they were still visible, while for the sample milled with the highest energy 5-PtNi/CeO_2_(+++), very weak Pt peaks were detected, indicating very small Pt NPs. In the samples 1-PtNi/CeO_2_(−–+) and 2-PtNi/CeO_2_(−+−), narrow metallic Ni reflections were also visible. The diffractogram of 5-Pt/CeO_2_(+++) exhibited only CeO_2_ reflections due to the very small size of Pt species (Figure S1). The monometallic 5-Ni/CeO_2_(+++) and Ni/CeO_2_ IWI samples showed only the NiO peaks (Figure S1) together with the CeO_2_ pattern. Metallic Pt and NiO reflections were present in the bimetallic PtNi/CeO2 seq-IWI sample. In the coimpregnated sample PtNi/CeO_2_ co-IWI, NiO reflections could be identified and no Pt peaks, suggesting that Pt is very well dispersed.

**Figure 1:**
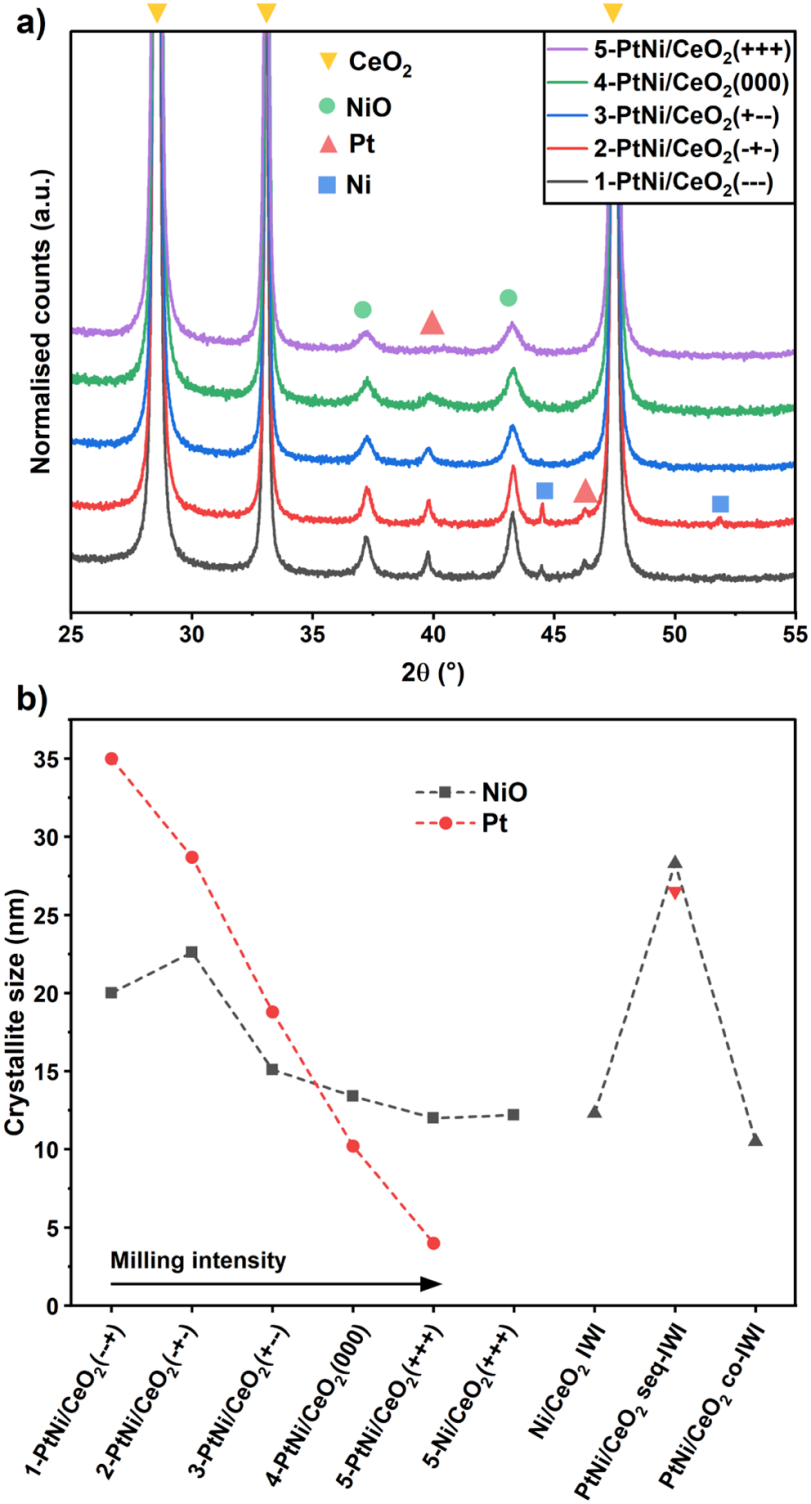
a) XRD patterns of PtNi/CeO_2_ catalysts after calcination at 400 °C for 4 h in air. b) NiO and Pt crystallite size calculated with the Scherrer equation.

The size of NiO, Pt, and Ni crystallites was estimated with the Scherrer equation using the full width at half-maximum (FWHM) of the NiO (200), Pt (111), and Ni (111) peaks (see Figure 1b, Table S3). There was a decreasing trend in the NPs’ average crystallite size with increasing milling intensity. The samples milled at 15 Hz, 1-PtNi/CeO_2_(−–+) and 2-PtNi/CeO_2_(−+−), showed the largest NiO NPs, measuring 20.0 and 22.6 nm, respectively, together with Pt NPs with sizes of 35.0 and 28.7 nm. The estimated size of the metallic Ni crystallites was larger than 100 nm. The NiO NP size decreased to 15.1 nm for 3-PtNi/CeO_2_(+–−) and 13.4 nm for 4-PtNi/CeO_2_(000), while the Pt size was 18.8 and 10.2 nm. Further size reduction was observed in the sample 5-PtNi/CeO_2_(+++) with a NiO size of 12.0 nm and a Pt size of less than 4 nm. The monometallic 5-Ni/CeO_2_(+++) sample, prepared using the same parameters as those for 5-PtNi/CeO_2_(+++), showed a comparable NiO NP size of 12.2 nm. Regarding the impregnated samples, Ni/CeO_2_ IWI showed NiO with a size of 12.3 nm, while for PtNi/CeO_2_ seq-IWI, large particles of NiO and Pt were detected, with sizes of 28.3 and 26.5 nm, respectively, possibly due to the double calcination for NiO and the scarce interaction of Pt with the preformed NiO. For PtNi/CeO_2_ co-IWI, the NiO size was 10.5 nm, due to a better interaction between Ni and Pt species and the single calcination step.

To investigate the effect of the milling forces on the bulk structure of ceria, the CeO_2_ crystallite size and lattice strain were estimated by Williamson–Hall (W–H) plot analysis. The W–H plots are shown in Figure S2, and the results of the analysis are reported in Table S4. The average CeO_2_ crystallite size was 52.9 ± 0.8 nm for the sample milled at the lowest intensity, which is similar to the value obtained for the impregnated samples, 53.2 ± 0.4 nm, both sequentially and coimpregnated. The CeO_2_ crystallite size slightly decreased by increasing the milling frequency to 47.5 and 44.2 nm for samples 4-PtNi/CeO_2_(000) and 5-PtNi/CeO_2_(+++), respectively. The residual lattice strain slightly decreased with increasing milling intensity, probably due to the additional energy provided to the lattice that helped relax more strain during the calcination. Overall, the wide range of milling intensities did not cause major differences in the bulk structure of the cerium oxide support.

### Temperature-Programmed Reduction

The H_2_ temperature-programmed reduction (H_2_-TPR) profiles of the PtNi/CeO_2_ samples synthesized with the DoE parameters are shown in Figure 2, while those for the monometallic and IWI samples are shown in Figure S3. For the monometallic 5-Ni/CeO_2_(+++) and Ni/CeO_2_ IWI samples, a single broad δ peak was present in the range from 260 to 400 °C, which could be decomposed into two Gaussian peaks centered at about 300 and 360 °C (Figure S3). The first peak is associated with the reduction of the NiO bulk, and the latter with the NiO in closer contact with CeO_2_, which is more difficult to reduce.^26,68^ For the milled 5-Ni/CeO_2_(+++) sample, the peak is slightly shifted to a higher temperature, indicating a stronger interaction with ceria. The monometallic 5-Pt/CeO_2_(+++) milled sample showed a single α peak at 130 °C associated with the reduction of Pt species in contact with ceria. This temperature is about 35 °C higher compared to values found by other authors for Pt/CeO_2_ catalysts prepared by impregnation,^26,69^ suggesting that the milling preparation created a stronger metal–support interaction.

**Figure 2:**
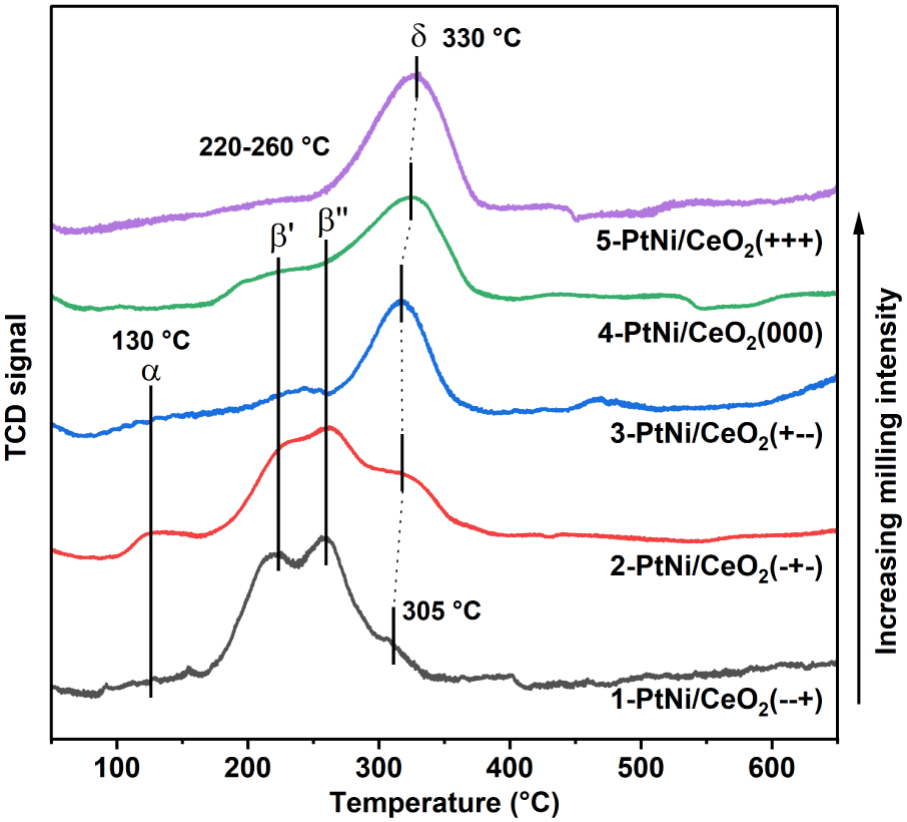
H_2_-TPR profiles of the DoE samples. The profiles are normalized and ordered by increasing milling energy.

In the bimetallic samples, three groups of peaks were detected: the α peak at 130 °C, corresponding to the reduction of oxidized Pt species on ceria; the β peaks at 220–260 °C, associated with the hydrogen spillover from metallic Pt to NiO;^27,70,71^ and the δ peaks at 300–360 °C, associated with the reduction of bulk NiO and NiO in contact with ceria.^26,68^ The bimetallic PtNi/CeO_2_ seq-IWI sample showed two main peaks centered at 220 and 305 °C, associated with the H_2_ spillover and the reduction of NiO. The same peaks were detected on the PtNi/CeO_2_ co-IWI sample, although the areas of the two peaks, β and δ, are inverted. This was probably due to the smaller size of NiO in the co-IWI sample, as estimated from XRD, where the amount of NiO in contact with CeO_2_ was higher. In addition, the δ peak was shifted to 310 °C, indicating a slightly stronger contact with ceria. Furthermore, the higher area of the β peak for PtNi/CeO_2_ seq-IWI could be ascribed to the sequential loading of Pt on the surface of already formed NiO/CeO_2_, increasing the amount of Pt exposed to H_2_ for the spillover effect.

Regarding the DoE samples (Figure 2), there was a trend within the β-to-δ area ratios and the position of the δ peak maximum temperature with the milling energy. At lower milling frequencies, the β peaks related to H_2_ spillover showed larger areas compared to the NiO bulk reduction peak, while they were less defined or almost disappeared at the highest milling frequencies. The area of the δ peaks grew with increasing milling energy, and the maximum temperature of the peak shifted from 305 to 330 °C, probably due to the smaller NiO size and increased interaction with CeO_2_ generated during the strong impacts. Compared with the monometallic 5-Ni/CeO_2_(+++) sample, the NiO reduction peak in 5-PtNi/CeO_2_(+++) occurred at lower temperatures owing to the addition of Pt.

### Transmission Electron Microscopy

The morphology and microstructure of 1-PtNi/CeO_2_(−–+), 5-PtNi/CeO_2_(+++), and PtNi/CeO_2_ seq-IWI were investigated by HRTEM. These samples are the sample synthesized with the highest milling frequency and BPR and longer milling times (5-PtNi/CeO_2_(+++)) and the sample prepared with the lowest milling frequency and BPR (1-PtNi/CeO_2_(−–+)), which represent the two opposite milling conditions. A bimetallic impregnated sample was also studied for comparison. Representative images are shown in Figure 3 and Figure S4. The sample 1-PtNi/CeO_2_(−–+), milled at 15 Hz for 5 min with a BPR of 20, was composed mainly of two types of NiO particles supported on CeO_2_, as shown in Figure 3a. Both NiO NPs with a size distribution of 11 ± 3 nm and agglomerates of large NiO particles with a size of more than 100 nm were identified. In Figure 3b, the EDX spectrum of the region marked in Figure 3a shows that the large agglomerates were mostly composed of NiO. No Pt NPs were clearly identified. Nevertheless, in Figure 3b and S4b weak EDX Pt signals over a large area of the catalyst were obtained, confirming the presence of Pt. This could be explained by the low loading of Pt in the sample and that it was present in a few large particles with a size of about 35 nm, as inferred from XRD (Table S3). This suggests a poor dispersion of Pt and consequently a low interaction between Pt and Ni at the lowest milling intensity. In PtNi/CeO_2_ seq-IWI, Pt clusters with a size of ca. 1 nm were observed to be highly dispersed over CeO_2_ and NiO crystallites (Figure 3c). The small Pt nanoclusters observed here are small enough to escape XRD detection. Similarly to 1-PtNi/CeO_2_(−–+), no Pt NPs were detected by TEM, possibly due to the low number of larger Pt NPs (size of ca. 26 nm as inferred from XRD, Table S3). In the sample 5-PtNi/CeO_2_(+++), small NiO nanoparticles in the proximity of Pt nanoclusters were identified. In addition, the NiO nanoparticles were slightly flattened (Figure 3e), indicating a strong interaction with CeO_2_, in agreement with H_2_-TPR measurements. The EDX spectrum of the area marked in Figure 3f (see inset) showed signals of both Ni and Pt from the surface of ceria decorated with small clusters.

**Figure 3:**
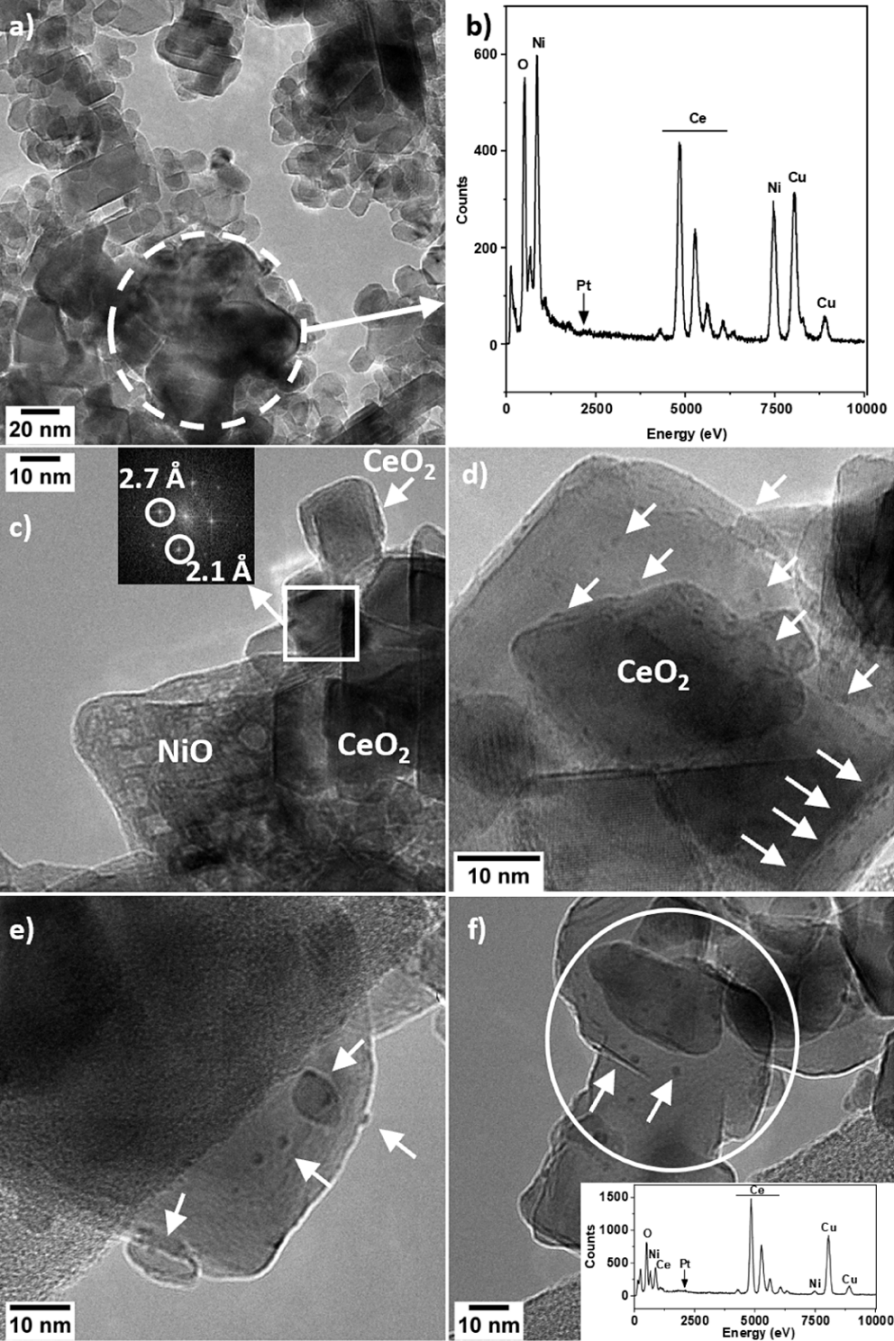
HRTEM images of a) 1-PtNi/CeO_2_(−–+), b) EDX spectrum of the region marked in a). c,d) PtNi/CeO_2_ seq-IWI; the spots at 2.1 Å in the Fourier transform correspond to NiO (200), while the ones at 2.7 Å correspond to CeO_2_ (200). e,f) 5-PtNi/CeO_2_(+++). The arrows indicate Pt nanoparticles.

### Catalytic Activity

The methane conversion between 700 and 950 °C of the DoE samples and the monometallic and IWI catalysts is reported in Figure 4a and b, respectively. In Figure 4c, the selectivity is shown. In all experiments, the only reaction products detected were H_2_, CO, and CO_2_. The products’ selectivity was similar for the different catalysts tested. For the milled samples, the methane conversion increased with an increase in milling energy. Low methane conversion and poor stability were shown by the catalysts with the lowest milling frequencies, namely 1-PtNi/CeO_2_(−–+) and 2-PtNi/CeO_2_(−+−), and their activity decreased at the lowest temperatures while rapidly increasing above 800 °C. The samples 3-PtNi/CeO_2_(+–−) and 4-PtNi/CeO_2_(000), milled respectively at 50 and 32.5 Hz for 5 and 25 min, showed higher methane conversion values with similar catalytic activity. The sample milled with the highest energy, 5-PtNi/CeO_2_(+++) (milled at 50 Hz for 45 min), showed the highest activity with 83.5% CH_4_ conversion at 700 °C and reached full conversion at 850 °C. The performance of H_2_ and CO chemisorption experiments to determine the number of active sites for these catalysts is controversial, as the CO chemisorption would result in the formation of nickel carbonyl species, while in H_2_ chemisorption experiments, there will be hydrogen spillover from Pt toward the ceria support, an effect that cannot be separated from the H_2_ chemisorption on the metals. Therefore, in the SI, we present the activity data as a function of the particle sizes determined by XRD. Figure S5a shows the methane conversion of the milled catalysts as a function of the NiO and Pt particle sizes. The methane conversion achieved by 5-PtNi/CeO_2_(+++) is remarkably higher (83.5%) than that of 4-PtNi/CeO_2_(000) and 3-PtNi/CeO_2_(+–−) (59.9% and 60.2%, respectively), while their NiO particle sizes are similar: 12.6, 13.6, and 14.6 nm, respectively, as inferred from XRD. Therefore, the differences observed in methane conversion can not only be ascribed to a particle size effect but also to a different interaction between Ni, Pt, and CeO_2_ created during the milling process. This is also in line with the TPR profile of 5-PtNi/CeO_2_(+++) that suggested the strongest interaction between the metals and CeO_2_ among the milled samples tested. The monometallic 5-Ni/CeO_2_(+++) catalyst (Figure 4b) showed catalytic activity similar to that of 5-PtNi/CeO_2_(+++). The presence of Pt did not affect the methane conversion, as Ni is more active than Pt toward the MSR reaction.^5,18^ At low temperatures, 5-Pt/CeO_2_(+++) showed a methane conversion comparable to that of the bimetallic impregnated samples with a stable performance at all temperatures, but lower compared to the monometallic Ni, as expected from the lower MSR catalytic activity of Pt compared to Ni.^5^ As shown in Figure S6, this catalyst had an induction period of more than 1 h, during which the methane conversion rate increased from 75 to 100 mol_CH4_ g_Pt_^–1^ h^–1^. For comparison, the methane conversion rate of 5-PtNi/CeO_2_(+++) normalized by the Pt loading reached the value of 186.2 mol_CH4_ g_Pt_^–1^ h^–1^, revealing the Ni role in the MSR performance.

**Figure 4:**
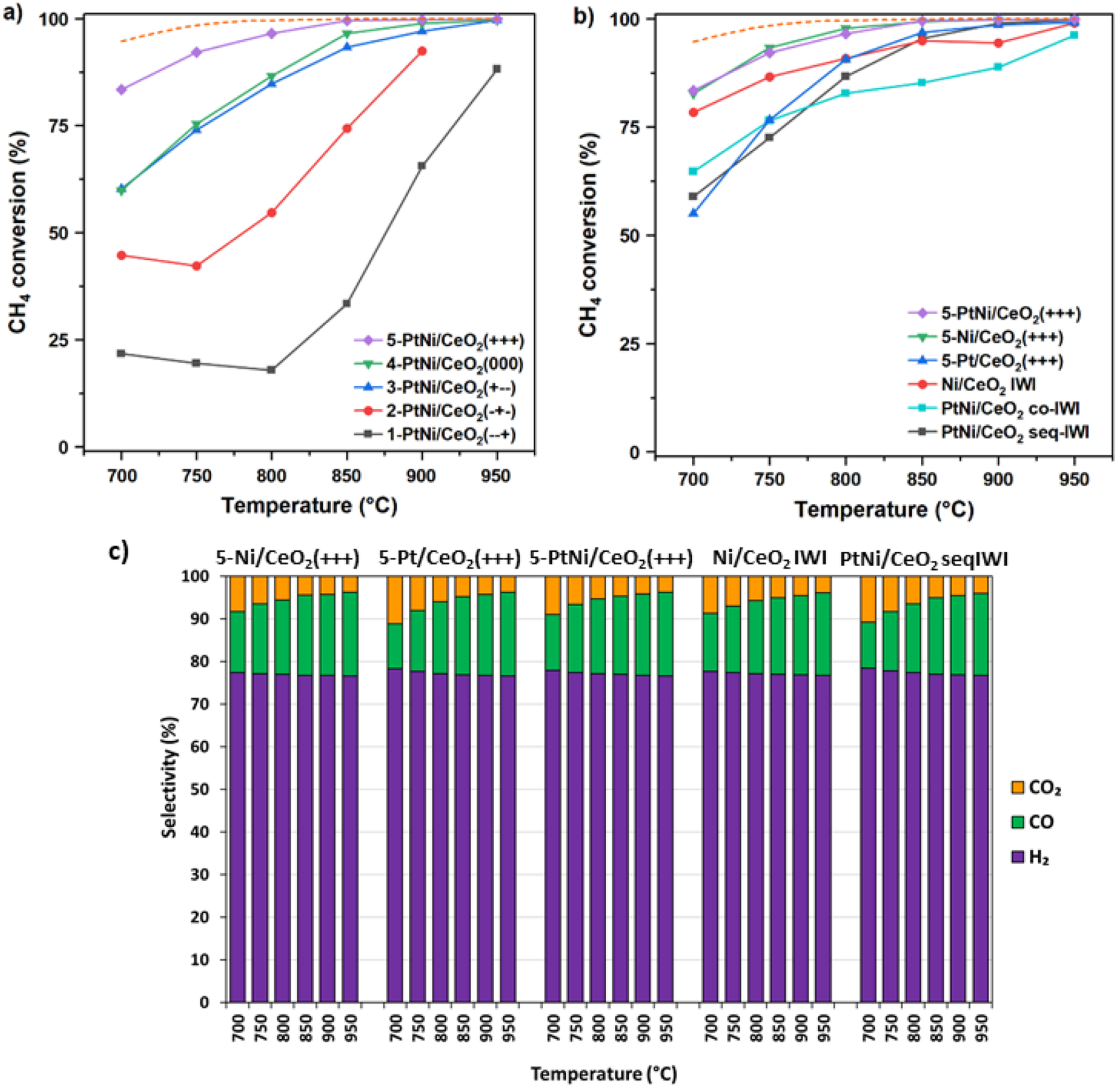
a) Methane conversion of the catalysts prepared from the DoE synthesis parameters. b) Methane conversion of the monometallic milled samples, IWI catalysts, and the best bimetallic PtNi/CeO_2_ milled catalyst. c) Selectivity corresponding to the reaction tests. Steam-to-carbon = 2, atmospheric pressure, and F/W = 202.500 mL g_cat_^–1^ h^–1^. The dashed line corresponds to the equilibrium conversion.

Regarding the IWI samples (Figure 4b), the bimetallic PtNi/CeO_2_ seq-IWI showed an activity comparable to the activity of 3-PtNi/CeO_2_(+–−) and 4-PtNi/CeO_2_(000). This sample exhibited a large size of the NiO NPs, which formed during the double calcination steps and resulted in a poor interaction between Ni and CeO_2_, as observed by TEM and H_2_-TPR. It is interesting to note that 5-Ni/CeO_2_(+++) showed a higher methane conversion than Ni/CeO_2_ IWI when normalized per gram of Ni (Figure S6 and Figure 4), despite the same NiO NP size being inferred from XRD. In Figure S5b, the methane conversion of mono- and bimetallic impregnated samples, as well as that of the best milled samples, is plotted as a function of the NiO and Pt particle sizes. In this plot, it is clear that methane conversion does not follow a linear trend with the NiO and Pt particle sizes. Catalyst PtNi/CeO_2_ co-IWI is the sample with the smallest NiO particle size (10.5 nm) and reaches 64% conversion, while 5-PtNi/CeO_2_(+++) reaches 83.5% methane conversion under the same reaction conditions, and the NiO particle size inferred from XRD is slightly larger (12.5 nm) than in PtNi/CeO_2_ co-IWI. This indicates that during the milling process, a distinct interaction between the metals and the support is created, which results in a different interaction. This is studied in the following sections.

The stability of the best bimetallic milled catalyst (5-PtNi/CeO_2_(+++)), the monometallic milled Ni and Pt catalysts, and the impregnated catalysts was evaluated (Figure S7a). The most active and stable catalysts were 5-PtNi/CeO_2_(+++) and 5-Ni/CeO_2_(+++). The milled bimetallic catalyst reached a higher methane conversion and showed a more stable performance than the impregnated counterparts. For the monometallic ones, the methane conversion of 5-Ni/CeO_2_(+++) showed a smaller deactivation rate than Ni/CeO_2_ IWI. Therefore, the milled catalysts showed an improved stability compared to that of the respective impregnated samples. Thermogravimetric analyses (TGA) were performed on the samples after the stability tests, and they are shown in Figure S7b. The weight variation observed for all catalysts is limited. For the Ni-containing samples, an initial weight loss is observed, corresponding to the loss of adsorbed water. After this weight loss, an increase is observed starting at ca. 200 °C, which corresponds to a weight gain due to the oxidation of Ni species. This increase is not observed in 5-Pt/CeO_2_(+++). After this increase in the weight, all samples keep constant the same weight, but 5-Ni/CeO_2_(+++), which features a slight decrease in the weight (from 101.2% to 100.1%) from 400 to 600 °C, which can be ascribed to a weight loss due to the decomposition of carbonaceous species. This indicates that limited carbon deposition took place in catalyst 5-Ni/CeO_2_(+++) during the stability test and that undetectable carbon deposition occurred in the other samples tested.

The DoE approach allows for estimating the effect of each milling parameter on the catalysts’ structure and activity.^10,72^ The responses chosen in this study were the NiO NP size, as smaller Ni particles are related to both higher reforming activity and catalyst stability,^10,73^ and the methane conversion achieved at 700 °C, as high methane conversions at low temperatures are pursued. The in-depth analysis of the correlation between methane conversion and the NP size with the milling parameters is provided in the SI (see also Figure S8), where, even though no statistical metrics could be calculated as more replicas would be needed, clear trends have been obtained. Analysis of the DoE trends shows that the most influential parameter in increasing the CH_4_ conversion and achieving a smaller NiO particle size is the milling frequency.

After screening the effect of the milling parameters and finding the parameters that lead to higher catalytic performances, an in-depth investigation of the structure of the catalysts showing the best performance, namely, 5-PtNi/CeO_2_(+++) and 5-Ni/CeO_2_(+++), was carried out. In this study, the IWI samples were included as a reference to unravel the changes induced by the milling process.

### Raman Spectroscopic Analysis of the Materials’ Structure

Raman spectroscopy was used to study the interaction among NiO, CeO_2_, and Pt in the best catalysts. In Figure 5a, the Raman signals of monometallic and IWI samples, as well as of a milled CeO_2_ support, are shown for comparison. In Figure 5b, the average Raman signals of the BM samples prepared with the best parameters are shown. Figure S10 shows the Raman spectra of all the milled samples. The signal of NiO, obtained from the decomposition of Ni­(Ac)_2_ calcined at 500 °C in air, is reported as a reference in Figure S9. Cerium oxide has a very distinctive Raman spectrum characterized by an intense and sharp peak at about 464 cm^–1^ (F2g) with an asymmetric shape at lower Raman shift, a broad feature at 1170 cm^–1^ (2LO), a weak peak at 250 cm^–1^ (2TA, corresponding to ceria surface defects and −OH terminations^74^), and the typical structure at 500–600 cm^–1^ associated with bulk and surface oxygen vacancies.^75^ To check the effect of high-energy milling on the CeO_2_ structure, the ceria support was milled with the same parameters as the best catalyst 5-PtNi/CeO_2_(+++), and the Raman spectrum is shown in Figure 5a. The analysis of the milled ceria revealed that the intensity of the bands at 500–600 cm^–1^ associated with defects and oxygen vacancies was very weak, representing 1% of the intensity in the normalized spectrum. The Raman signal of the unmilled CeO_2_ support is also shown in Figure 5a, and it is very similar to the spectrum of the milled ceria, with the normalized intensity of the defect bands being about 1% as well. This indicates that milling at the highest energy was not enough to directly induce defects and oxygen vacancies in the ceria structure, in accordance with the W–H plot analysis shown in Figure S2.

**Figure 5:**
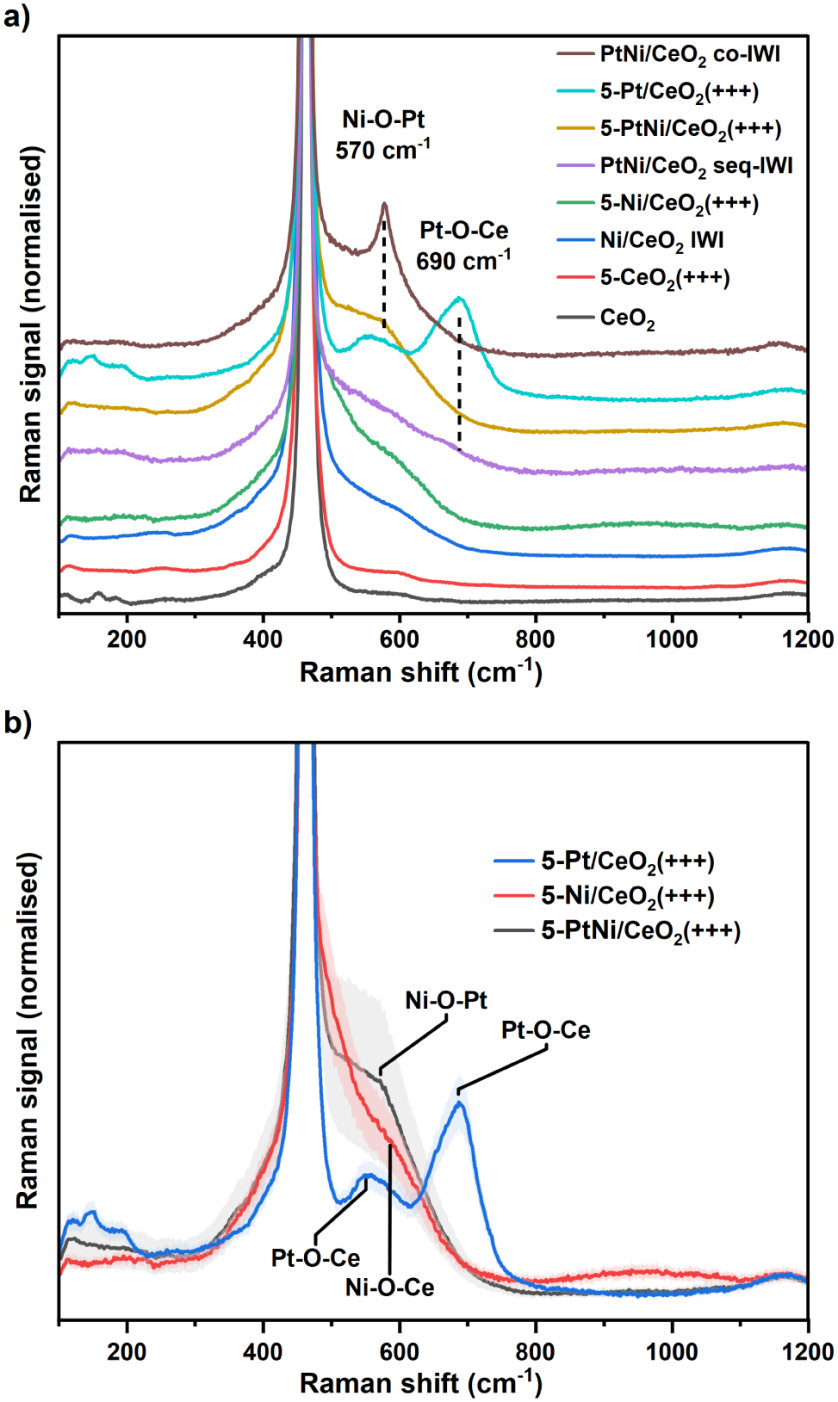
Normalized Raman spectra of a) reference materials, monometallic catalysts, 5-PtNi/CeO_2_(+++), and IWI catalysts; b) the catalysts prepared with the parameters leading to the highest methane conversion found with the DoE analysis. The bands around the spectra represent the standard deviation of the 7–11 spectra averaged. Wavelength = 532 nm, power = 1 mW, and objective = 50×.

The next structure to consider is the interaction between NiO and CeO_2_. In the monometallic 5-Ni/CeO_2_(+++) and Ni/CeO_2_ IWI samples, this interaction gave rise to a broad band centered around 590 cm^–1^ and a more pronounced asymmetry at 400 cm^–1^.^76^ The intensity of the band at 590 cm^–1^ is higher for the milled sample, possibly indicating higher interaction with CeO_2_, as seen in the TPR (Figure S3). The Raman signal from the monometallic 5-Pt/CeO_2_(+++) sample showed the typical spectrum of Pt/CeO_2_ samples, with two broad asymmetric bands centered at 550 and 690 cm^–1^ associated with the Pt–O–Ce bond vibrations.^77,78^ In addition, three small bands were observed at 120–200 cm^–1^.

Regarding the bimetallic catalysts, the spectrum of PtNi/CeO_2_ co-IWI showed a sharp and intense feature at 570 cm^–1^, not present in the monometallic samples, which we tentatively relate to a strong Ni–O–Pt interaction. The band at 690 cm^–1^, typical of the Pt–O–Ce bond, is completely missing, together with the three weak bands at 120–200 cm^–1^, suggesting that Pt is interacting mostly with NiO. The Raman signal of 5-PtNi/CeO_2_(+++) is characterized by an intense band between 500 and 600 cm^–1^ featuring also a shoulder at 570 cm^–1^, which seems to further prove that this feature is related to the interaction between NiO and Pt species, as inferred from TPR. On the other hand, the average signal from PtNi/CeO_2_ seq-IWI shows a broad band between 500 and 600 cm^–1^, resembling that of the monometallic samples and thus corresponding to NiO–Ce interaction, as expected from the scarce interaction between NiO and the isolated Pt NPs observed by TEM (Figure 3c,d).

The direct comparison between the best-milled samples shown in Figure 5b highlights the different shapes of the defective band at 500–600 cm^–1^ associated with Pt–NiO interactions. The F2g peak of CeO_2_ remained at 464 cm^–1^ for all the samples measured in this study, indicating that no solid solution of NiO in CeO_2_ was formed.^79^ At the same time, the FWHM of the F2g peak remained constant with values in the range of 11.5–12.0 cm^–1^ for all the samples considered in this study, corresponding to CeO_2_ crystals of 60–75 nm, in agreement with the XRD characterization and suggesting that no bulk modifications were introduced during the milling.^80,81^ In Figure S10, the Raman spectra of the DoE samples are shown, highlighting the increasing NiO–CeO_2_ and Pt–NiO interactions with higher milling energy.

### *In Situ* X-ray Absorption Spectroscopy

The structure of Ni in 5-PtNi/CeO_2_(+++), 5-Ni/CeO_2_(+++), and PtNi/CeO_2_ seq-IWI was studied element-specifically by recording *in situ* XANES spectra at the Ni K-edge during the reduction in H_2_ up to 700 °C and during the MSR reaction in a light-off experiment up to 700 °C. EXAFS spectra were acquired at room temperature for the as-prepared samples, after reduction in H_2_ up to 700 °C, and after MSR. They are shown in Figure 6. The EXAFS data were fitted, and the results are presented in Table S6 and Figure S11.

**Figure 6:**
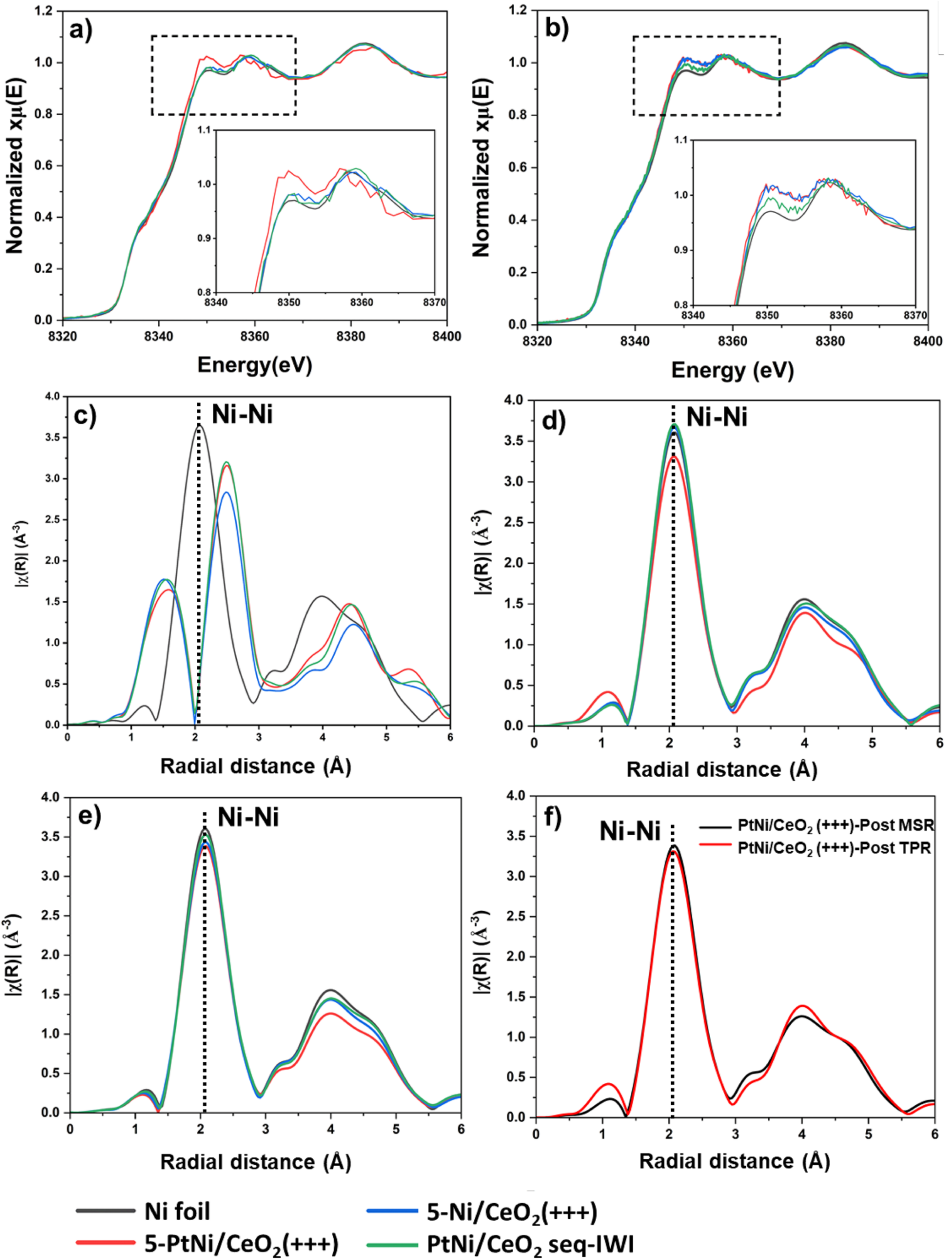
Ni K-edge XANES spectra collected at room temperature under He of samples 5-PtNi/CeO_2_(+++), 5-Ni/CeO_2_(+++), and PtNi/CeO_2_ seq-IWI. a) After the TPR at 700 °C. b) After the MSR tests at 700 °C (H_2_O + CH_4_ at S/C = 2). The FT magnitude of the Ni K-edge EXAFS spectra of c) as-prepared catalysts. d) After the TPR step. e) After MSR. f) Comparison of the FT magnitude of 5-PtNi/CeO_2_(+++) after TPR and MSR. The Ni foil is also shown for comparison.

A linear combination analysis of the XANES spectra during the reduction up to 700 °C (see Figure S12) showed that the onset temperatures for the reduction of nickel oxide species to Ni, corresponding to the temperature where already 10% metallic Ni was present, were 206, 199, and 260 °C for 5-Ni/CeO_2_(+++), 5-PtNi/CeO_2_(+++), and PtNi/CeO_2_ seq-IWI, respectively. After each step, the samples were cooled to RT in He. In Figure 6, the Ni K-edge XANES spectra are reported. After the reduction treatment, once the samples were cooled to RT, all the samples resembled the Ni foil spectrum, except 5-PtNi/CeO_2_(+++), which showed a more intense white line at 8350 eV (Figure 6b). This has been observed in Pt–Ni alloys with a low content of Pt, and it has been associated with the electronic donation from Ni to the more electronegative Pt in the alloy.^17,82–84^ The bimetallic PtNi/CeO_2_ seq-IWI showed the same profile as 5-Ni/CeO_2_(+++), suggesting scarce interaction between Pt and Ni and the large size of Pt and Ni NPs.

During the methane steam reforming reaction at 700 °C for 1 h, Ni was maintained fully reduced in all samples. After 1 h of reaction, the samples were cooled in He. The absorption edge of PtNi/CeO_2_ seq-IWI was very similar to that after the reduction (see Figure 6b and its inset), as expected from the presence of large Ni particles. Instead, the two milled samples showed different spectra after MSR. The bimetallic 5-PtNi/CeO_2_(+++) catalyst showed a XANES spectrum very similar to that of 5-Ni/CeO_2_(+++). Hence, the feature associated with the Pt–Ni interaction disappeared.^21,85,86^ The XANES spectrum of 5-Ni/CeO_2_(+++) after MSR is different compared to the one after TPR, as the feature at 8350 eV is more intense. Probably, this can be explained by the formation of a strong metal–support interaction, typical for reduced CeO_2‑x_ and Ni, and the interface Ni–CeO_2‑*x*_, which was identified as the active site for MSR by Salcedo et al.^9^ The similarities between the XANES spectra of the milled samples can be explained by the presence of similar material structures, such as monometallic Ni particles interacting with reduced CeO_2‑*x*_ for the segregation of Pt in the bimetallic catalyst.

The FT EXAFS spectra of the as-prepared catalysts show the presence of fully oxidized Ni species (Figure 6c). After the TPR step, all samples were completely reduced and all showed the typical features of metallic Ni. In the case of the 5-PtNi/CeO_2_(+++), a slightly lower Ni–Ni coordination number (CN) was obtained than for the other catalysts, indicating smaller particles. In Table S6, the values of the fits are reported. After MSR, again all catalysts show a similar structure to that of the Ni foil, and only small differences are observed for higher *R* values (3–5 Å) for the milled bimetallic catalyst. Comparing the spectra of this catalyst before and after MSR at 700 °C, almost no differences were observed, and the CN amounted to 9.5 ± 1.4 and 9.8 ± 1.0, respectively, indicating that hardly any sintering occurred for this sample during the reaction and the nanoparticles kept their initial size.

### *In Situ* Near-Ambient Pressure X-ray Photoelectron Spectroscopy

The surfaces of 5-PtNi/CeO_2_(+++) and 5-Ni/CeO_2_(+++), which are the most active catalysts, were further studied by *in situ* NAP-XPS. The spectra of the Ce 3d and Ni 2p regions were measured under different atmospheres and temperatures at 1 mbar of pressure with a kinetic energy (KE) of 215 eV (IMFP ≈ 0.6 nm, corresponding to the topmost surface layers, Table S1) and are shown in Figure 7 for both catalysts. The same region, measured at KE = 450 eV (IMFP ≈ 1 nm, with a higher contribution of the subsurface layers), is shown in Figure S13. The degree of reduction of CeO_2_, the metal/support atomic ratios, Pt/Ni atomic ratios, and the Ce, Pt, and Ni atomic concentrations are summarized in Figure 8 and Table S7. The spectra of Pt 4f + Ni 3p and O 1s are shown in Figures S14 and S15.

**Figure 7:**
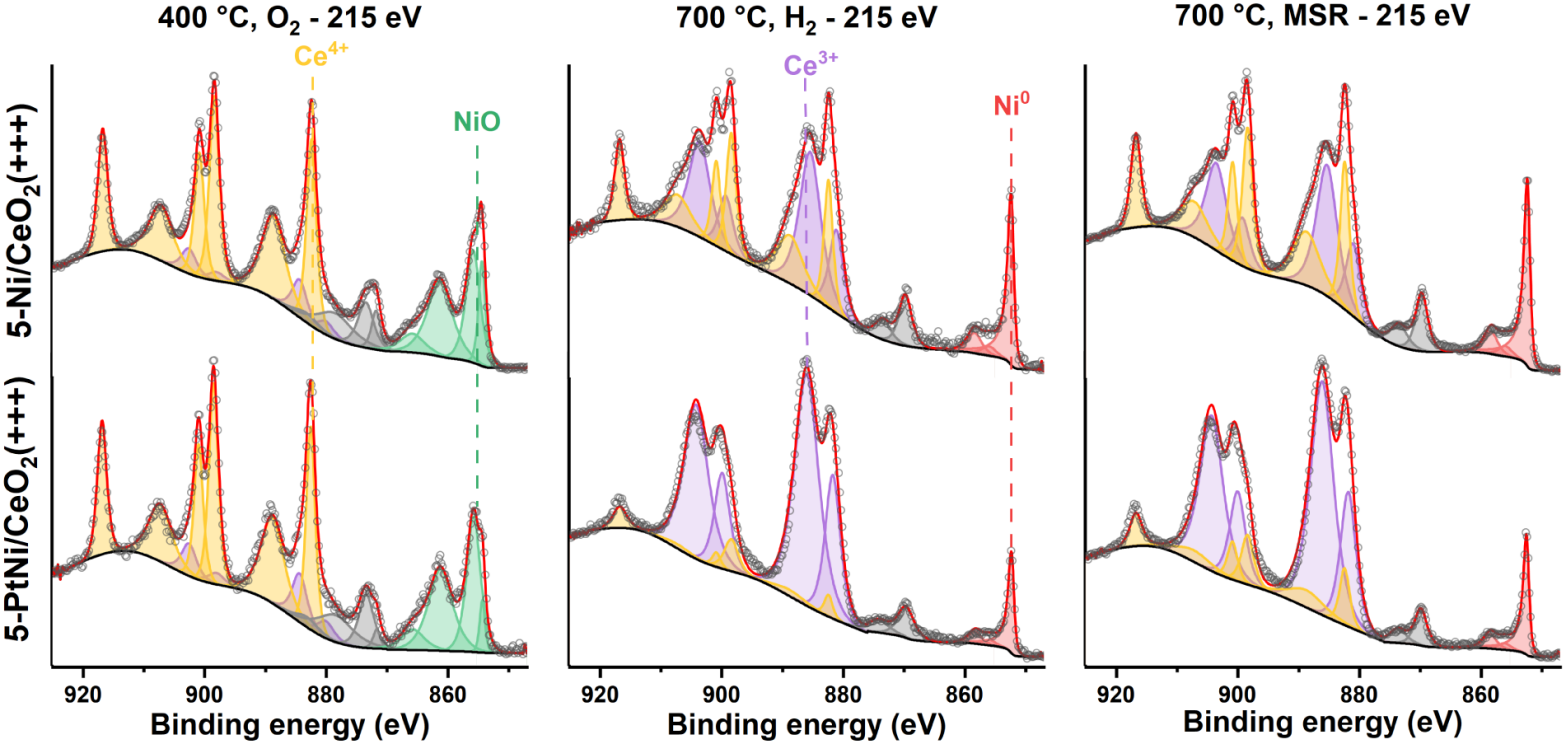
NAP-XPS spectra of the Ce 3d and Ni 2p regions of catalysts 5-Ni/CeO_2_(+++) and 5-PtNi/CeO_2_(+++) measured at a kinetic energy of 215 eV under the atmospheres and temperatures indicated (total pressure = 1 mbar). The spectra are normalized.

**Figure 8:**
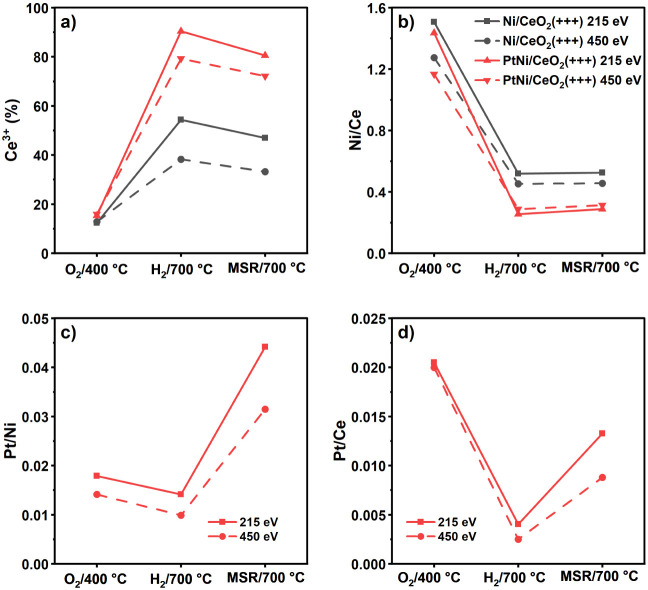
NAP-XPS results summary for catalysts 5-PtNi/CeO_2_(+++) and 5-Ni/CeO_2_(+++) at 1 mbar under the indicated atmospheres and temperatures. a) Percentage of reduced Ce^3+^ on the catalyst surface, b) Ni/Ce atomic ratio, c) Pt/Ni atomic ratio for 5-PtNi/CeO_2_(+++), and d) Pt/Ce atomic ratio for 5-PtNi/CeO_2_(+++).

Under O_2_ at 400 °C, the surface of both catalysts was fully oxidized: the main peak of the Ni 2p_3/2_ region was at 855 eV, with a shape corresponding to a mixture of NiO and Ni­(OH)_2_.^64^ For both catalysts, the amount of Ce^3+^ on the surface was ca. 15% (Figure 8a). The Ni/Ce ratio was 1.5 and 1.4 for 5-Ni/CeO_2_(+++) and 5-PtNi/CeO_2_(+++) at 215 eV, respectively, and 1.3 and 1.2 at 450 eV. For 5-PtNi/CeO_2_(+++), the Pt/Ni atomic ratio was 0.018 and 0.014 at 215 and 450 eV, respectively, indicating a slightly enriched Pt surface. The O 1s region showed two main peaks centered at 529.7 and 531.5 eV, corresponding to the lattice oxygen of CeO_2_ and NiO and the possible contribution of adsorbed hydroxyl species (Figure S15).^64,87^ In this region, the doublet of gaseous O_2_ was also visible at 538.5 eV.

After changing the atmosphere to H_2_ and increasing the temperature to 700 °C, the oxidized Ni species got completely reduced to metallic Ni (BE = 852.6 eV),^64^ and Pt was reduced to metallic Pt (BE = 71.2 eV).^88^ The spectrum of ceria showed distinct signs of reduction. In particular, the degree of CeO_2‑*x*_ reduction in 5-Ni/CeO_2_(+++) was 54% and 38% at KE values of 215 and 450 eV, while in 5-PtNi/CeO_2_(+++) the degree of reduction was much higher: 90% and 79% at the surface and subsurface regions. The presence of Pt favored the hydrogen spillover from the metallic nanoparticles to the support surface more strongly than Ni, leading to a more reduced ceria surface.^89–92^ The Ni/Ce ratio dropped to 0.52 (KE = 215 eV) and 0.45 (KE = 450 eV) for 5-Ni/CeO_2_(+++) corresponding to a decrease of 65% compared with the sample under oxygen, while for 5-PtNi/CeO_2_(+++) the Ni/Ce ratios were 0.25 (KE = 215 eV) and 0.29 (KE = 450 eV), representing a drop of 82% and 75%, respectively. The Pt/Ni ratio decreased slightly both on the surface and subsurface regions, remaining similar to the oxidized sample, and the Pt/Ce ratio decreased similarly to the Ni/Ce ratio for 5-PtNi/CeO_2_(+++). The decrease in the Ni/Ce and Pt/Ce ratios can be ascribed to several factors: (i) under H_2_ at 700 °C, the metal NPs agglomerate, causing the decrease of the metals/Ce ratio. (ii) The high reduction degree of the ceria surface suggests that the Ni NPs were partially covered by the ceria surface due to the strong metal–support interaction (Figure 8b) and promoting a higher Ni/Ce ratio deeper in the bimetallic catalyst’s surface.^42,69^ The O 1s region reflected the changes observed on Ni and Ce (Figure S15): the peaks centered at 531.5 eV related to NiO species disappeared as NiO reduced to Ni, and a new peak at 530.8 eV related to O–Ce^3+^ appeared.^87^ The intensity of this peak is related to the amount of Ce^3+^ on the surface of the catalysts, and it is more intense for 5-PtNi/CeO_2_(+++). A second isolated peak at 535.8 eV related to gaseous H_2_O was also observed during the reduction step (also detected by the mass spectrometer, Figure S16),^93^ while bands centered around 532.6 eV could be associated with adsorbed water.^64^

Upon dosing the MSR mixture at 700 °C (CH_4_ and H_2_O at S/C = 2), H_2_, CO, and CO_2_ were produced in both samples, as observed with the mass spectrometer, confirming that the catalysts were active during the measurements (Figure S16). Additionally, peaks in the range of 535 and 538 eV in the O 1s region, related to H_2_O and CO_*x*_ in the gas phase, were observed on the samples’ surface.^93–95^ In both samples, Ni and Pt did not change and remained metallic (Figures 7, S13, and S14). The ceria surface was slightly oxidized, although the surface of 5-PtNi/CeO_2_(+++) maintained a higher degree of reduction compared to 5-Ni/CeO_2_(+++) (80% vs 47% of Ce^3+^, respectively, at KE = 215 eV). The Ni/Ce ratio remained constant for 5-Ni/CeO_2_(+++), while it slightly increased at the surface for 5-PtNi/CeO_2_(+++) (Figure 8b). Interestingly, a reorganization of Pt and Ni was observed in 5-PtNi/CeO_2_(+++): the Pt/Ni ratio drastically increased from 0.014 and 0.010 under H_2_, to 0.044 and 0.031 under MSR (respectively at 215 and 450 eV, Figure 8c,d), indicating a strong segregation of Pt toward the catalyst’s surface, in agreement with the weaker interaction between Ni and Pt observed by XANES (see Figure 6c,d). Indeed, while the Ni/Ce ratio remained almost constant, the Pt/Ce ratio increased by 230–250% upon changing to MSR conditions, with the Pt concentration in both the surface and subsurface regions being close to the nominal loading. This result has been observed with other techniques and is associated with higher coke resistance by other authors.^21,27,85,86^ This indicates that Pt segregation and/or redispersion on the surface and subsurface regions decreases carbon deposition. The O 1s spectra showed similar contributions to the samples under H_2_. An increase in the peak at 530.9 eV was observed due to the higher coverage of adsorbed −OH species due to the formation of H_2_O during MSR.^39^

Finally, the C 1s region of the samples under the MSR is shown in Figure 9. Together with the peak from Ce 4s (291 eV), the gas-phase peaks related to CH_4_, CO, and CO_2_ were detected.^94–96^ Most interestingly, monometallic 5-Ni/CeO_2_(+++) distinctly showed the presence of carbon on the surface at 285 eV, while the surface of 5-PtNi/CeO_2_(+++) was completely clean of carbon species under the same conditions. This remarkable result proves that Pt addition undoubtedly leads to less coke formation, which leads to more stable catalysts. These two samples were analyzed by Raman spectroscopy after the stability tests, and the Raman spectra of 5-Ni/CeO_2_(+++) showed peaks corresponding to coke, which were absent in the case of 5-PtNi/CeO_2_(+++), in line with the observations made by NAP-XPS (Figure S17).

**Figure 9:**
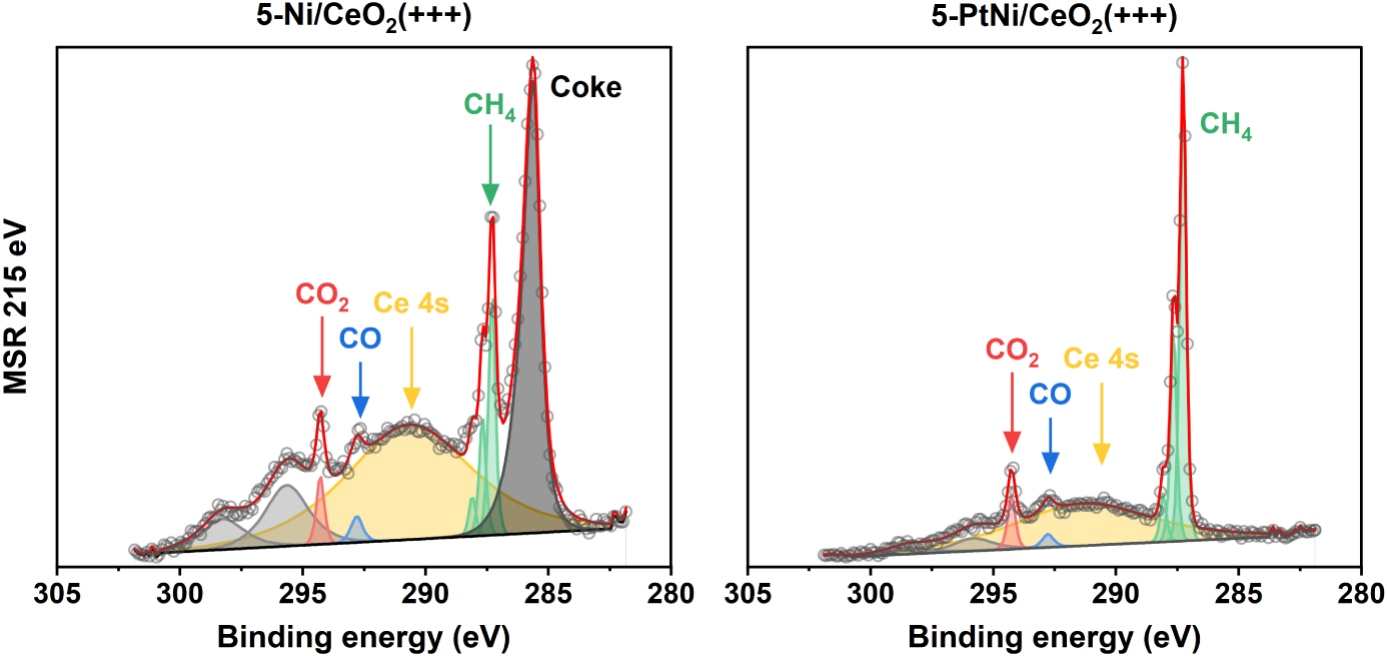
NAP-XPS spectra of C 1s regions of 5-Ni/CeO_2_(+++) and 5-PtNi/CeO_2_(+++) under MSR (S/C = 2, T = 700 °C, KE = 215 eV). Differences in the relative intensities of the gas phase peaks and Ce 4s are due to the different positioning of the XPS nozzle during the two experiments.

## Conclusions

In this work, we performed a fractional factorial design of an experiment to rationalize the synthesis of bimetallic PtNi/CeO_2_ catalysts prepared by ball milling. With this methodology, we could efficiently assess the effect of the milling conditions, namely frequency, milling time, and ball-to-powder ratio, on the structure and activity of the catalysts toward methane steam reforming. The DoE analysis revealed that the most influential parameter to achieve higher methane conversion rates and smaller NiO particles was the milling frequency, as the milling energy is proportional to the cube of the milling frequency, while longer milling times increased the milling energy to a minor extent. XRD, H_2_-TPR, and Raman spectroscopic analysis revealed that by increasing the milling energy on a mass basis, the catalyst structure was modified so that stronger interactions between the ceria support and the metal nanoparticles were obtained. These structural changes occurring during milling led to higher catalytic performances, with the milled catalysts achieving the highest methane conversions and surpassing the performances obtained by the counterparts prepared by traditional impregnation on a mass basis. The best-performing milled catalysts were investigated by synchrotron *in situ* XANES and NAP-XPS. *In situ* XANES indicated the creation of different catalytic sites during milling: both Ni/CeO_2_ and PtNi/CeO_2_ milled catalysts, after the MSR tests, showed features that can be related to a strong metal–support interaction, typical of reduced CeO_2‑*x*_ and Ni, and the Ni–CeO_2‑*x*_ interface, which were not present in the impregnated PtNi/CeO_2_ catalyst. *In situ* NAP-XPS measurements uncovered that the presence of Pt led to almost complete reduction of the ceria surface during the reduction and MSR, which is very reactive toward H_2_O activation and coke gasification. During MSR, a strong Pt segregation toward the surface was observed, and remarkably, no coke formation was detected during the *in situ* NAP-XPS measurements in the bimetallic PtNi/CeO_2_ catalyst. These observations are in contrast to the results of the monometallic Ni/CeO_2_ catalyst, where strong carbon signals related to solid coke were recorded on the surface. This result was reproduced in packed bed reactors in a 10-h stability test and confirmed by Raman spectroscopic analyses, where no C signals were detected in the bimetallic catalyst.

## Supplementary Material



## Notes

Supplementary tables and figures; XRD and DoE analyses (PDF)
