# Supplementary material for: Mechanochemically Engineered Bimetallic PtNi/CeO2 Catalysts for Enhanced Methane Steam Reforming
Source: ACS Catal. 2026 Feb 4;16(4):3159–74. doi: 10.1021/acscatal.5c06508 (PMC12930346; doi:10.1021/acscatal.5c06508)
Supplement: Supplementary file 1 [file cs5c06508_si_001.pdf]

Supporting information for: Mechanochemically Engineered Bimetallic PtNi/CeO_2 Catalysts for Enhanced Methane Steam Reforming.

Andrea Braga[1], Marina Armengol-Profitós[1], Laia Pascua-Solé[1], Lluís Soler[1], Isabel Serrano[1], Ignacio J. Villar-Garcia[2], Virginia Pérez-Dieste[2], Enrico Tusini[3], Andrea De Giacinto[3], Anna Zimina[4], Jan-Dierk Grunwaldt[3,4], Jordi Llorca[1*], Núria J. Divins[1*]

[1] Department of Chemical Engineering, Institute of Energy Technologies, and Center for Research in Multiscale Science and Engineering, Universitat Politècnica de Catalunya, EEBE, Eduard Maristany 10-14, 08019 Barcelona, Spain

[2] ALBA Synchrotron Light Source, Carrer de la Llum 2-26, 08290 Cerdanyola del Vallès Barcelona, Spain

[3] Institute for Chemical Technology and Polymer Chemistry, Karlsruhe Institute of Technology (KIT), Engesserstraße 20, 76131 Karlsruhe, Germany

[4] Institute of Catalysis Research and Technology, Karlsruhe Institute of Technology (KIT), Hermann-von-Helmholtz-Platz 1, 76344 Eggenstein-Leopoldshafen, Germany

[*] e-mail: nuria.jimenez.divins@upc.edu, jordi.llorca@upc.edu

Table S1. Inelastic mean free path values of electrons emitted at kinetic energies of 215 and 450 eV. Values are obtained from the NIST IMFP database using the Tanuma, Powell and Penn entries. [1]

Spectral region	IMFP - 215 eV (nm)	Photon energy (eV)	IMFP - 450 eV (nm)	Photon energy (eV)
**Ce 3d**	0.7	1100	1.1	1335
**Ni 2p**	0.6	1100	0.9	1335
**Pt 4f**	0.5	305	0.8	532

Table S2. Catalyst compositions measured by ICP-OES.

Sample	Ni wt.%	Pt wt.%	Pt/Ni wt.%	Pt/Ni at.%
**1-PtNi/CeO_2(--+)**	7.8	0.9	0.11	0.034
**2- PtNi/CeO_2(-+-)**	7.9	1.0	0.13	0.038
**3- PtNi/CeO_2(+--)**	7.6	0.8	0.10	0.031
**4- PtNi/CeO_2(000)**	8.0	1.0	0.13	0.038
**5- PtNi/CeO_2(+++)**	7.9	1.0	0.13	0.038
**5-Ni/CeO_2(+++)**	7.6	0.0	-	-
**5-Pt/CeO_2(+++)**	0.0	1.1	-	-
**PtNi/CeO_2 seq-IWI**	9.0	1.0	0.11	0.033
**Ni/CeO_2 IWI**	7.9	0.0	-	-

Estimation of the milling energy

The milling system is a shaker mill type in which a single ball used in this study moves with a linear trajectory along the vertical direction covering a distance L while the jar is moving in the opposite direction. The impact velocity of the ball V_b is thus given by: [2]

$$V_b = \frac{2 \cdot L}{\Delta t} = 2 \cdot L \cdot \nu \quad (1)$$

where the time is related to the oscillation frequency (ν, *Hz*) of the jar.

The kinetic energy carried by the ball and transferred to the powder is given by:

$$E_k = k \, \frac{1}{2} \, m_b V_b^2 = 2 \, k \cdot m_b \cdot L^2 \nu^2 \quad (2)$$

where k is a constant accounting for the type of collision (k = 1 for inelastic collision, as in the case of the balls covered by a layer of powder [3]) and m_b is the mass of the ball.

The total energy exchanged during the milling process is connected to the number of impacts by:

$$E_{tot} = N_{impacts} \cdot E_k = 60 \cdot t \cdot \nu \cdot E_k \ (3)$$

where t is the milling time in minutes.

The energy transferred to a given amount of powder, m_p, is thus related to:

$$\frac{E_{tot}}{m_p} = \frac{N_{impacts} \cdot E_k}{m_p} = \frac{60 \cdot t \cdot \nu \cdot 2 \, k \cdot m_b \cdot L^2 \nu^2}{m_p} = 60 \cdot t \cdot \nu \cdot 2 \, k \cdot L^2 \nu^2 \cdot BPR \ (4)$$

where $BPR = m_b / m_p$.

Considering the oscillation length, the parameter k and other aspects of the milling process as constant among the different syntheses, overall, the milling energy by mass is proportional to:

$$milling\ energy\ by\ mass \propto t \cdot BPR \cdot \nu^3 \ [s^{-2}] \ (5)$$

XRD Characterisation

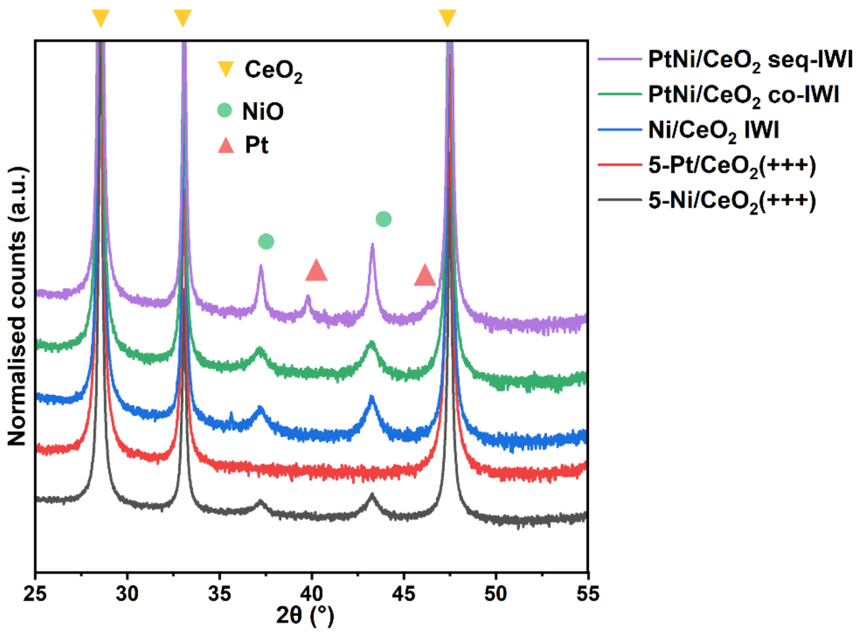

Figure S1. X-ray diffraction patterns of monometallic ball milled samples and IWI references.

Table S3. Crystallite size for NiO, Pt and Ni for mono- and bimetallic PtNi/CeO_2 samples, as calculated with Scherrer equation from the NiO (200) peak at 43.3°, Pt (111) peak at 39.8° and Ni (111) peak at 44.5°.

Sample	NiO (nm)	Pt (nm)	Ni (nm)
**1-PtNi/CeO_2(--+)**	20.0	35.0	> 100
**2-PtNi/CeO_2(-+-)**	22.6	28.7	> 100
**3-PtNi/CeO_2(+--)**	15.1	18.8	-
**4-PtNi/CeO_2(000)**	13.4	10.2	-
**5-PtNi/CeO_2(+++)**	12.0	< 4	-
**5-Ni/CeO_2(+++)**	12.2	-	-
**Ni/CeO_2 IWI**	12.3	-	-
**PtNi/CeO_2 seq-IWI**	28.3	26.5	-
**PtNi/CeO_2 co-IWI**	10.5	-	-

Williamson-Hall analysis

The width of XRD reflections is a convolution between the broadening related to the finite size of nanocrystals and the broadening induced by the residual strain, together with the instrumental broadening. [4] In the Williamson-Hall analysis, the width of each peak is expressed as $B = B_d + B_\epsilon$ where B_d is the contribution of the size broadening (equation 6) and B_ϵ is the strain broadening (equation 7):

$$B_d = \frac{K \cdot \lambda}{d \cdot \cos(\theta)} \quad (6)$$

$$B_\epsilon = 4 \cdot \epsilon \cdot \tan(\theta) \quad (7)$$

where $K = 0.9$ is the Scherrer shape factor, λ is the X-ray wavelength, d is the crystallite size, θ is the Bragg diffraction angle and ε is the residual lattice strain. By rearranging the two equations, it is possible to plot the

values *cos(θ)·FWHM* for each peak versus *4·sin(θ)* and to fit the data with a linear regression (equation 8), where the intercept of the line is related to the crystallite size, and the slope is related to the strain:

$$FWHM \cdot \cos(\theta) = \frac{K \cdot \lambda}{d} + 4 \cdot \epsilon \cdot \sin(\theta) = q + m \cdot x \ (8)$$

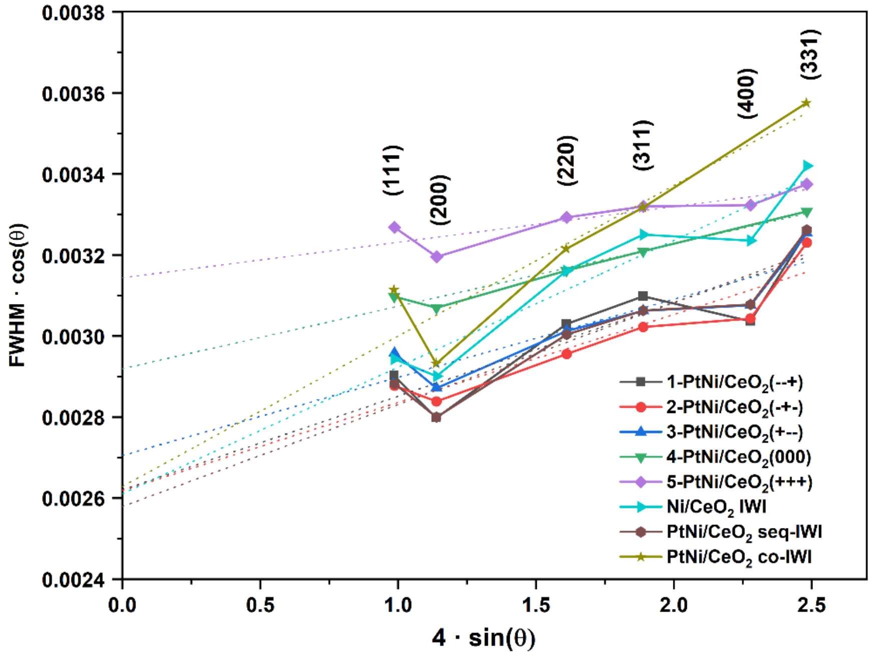

Figure S2. Williamson-Hall plots of the CeO_2 peaks of PtNi/CeO_2 samples and the corresponding linear fits.

Table S4. Linear fit parameters of the Williamson-Hall plot analysis, and calculated CeO_2 crystallite size and residual lattice strain.

Sample	Intercept	Slope	CeO_2 size (nm)	ε (%)
**1-PtNi/CeO_2(--+)**	0.0026	2.31E-04	52.9	0.23
**2-PtNi/CeO_2(-+-)**	0.0027	1.93E-04	51.4	0.19
**3-PtNi/CeO_2(+--)**	0.0026	2.17E-04	52.9	0.22
**4-PtNi/CeO_2(000)**	0.0029	2.02E-04	47.5	0.15
**5-PtNi/CeO_2(+++)**	0.0031	8.78E-05	44.2	0.09
**PtNi/CeO_2 seq-IWI**	0.0026	2.52E-04	53.7	0.25
**PtNi/CeO_2 co-IWI**	0.0026	3.71E-04	52.7	0.37
**Ni/CeO_2 IWI**	0.0026	3.13E-04	53.1	0.31

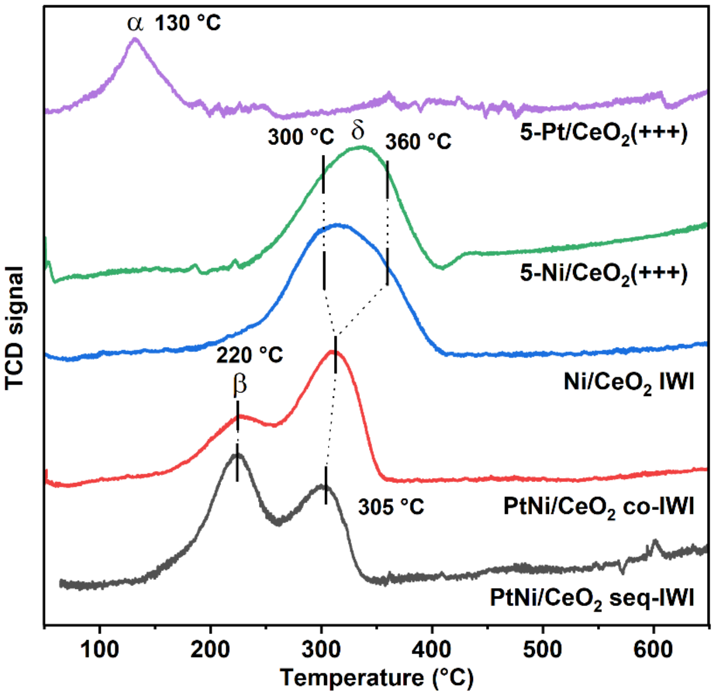

Figure S3. H_2-TPR of samples outside DoE.

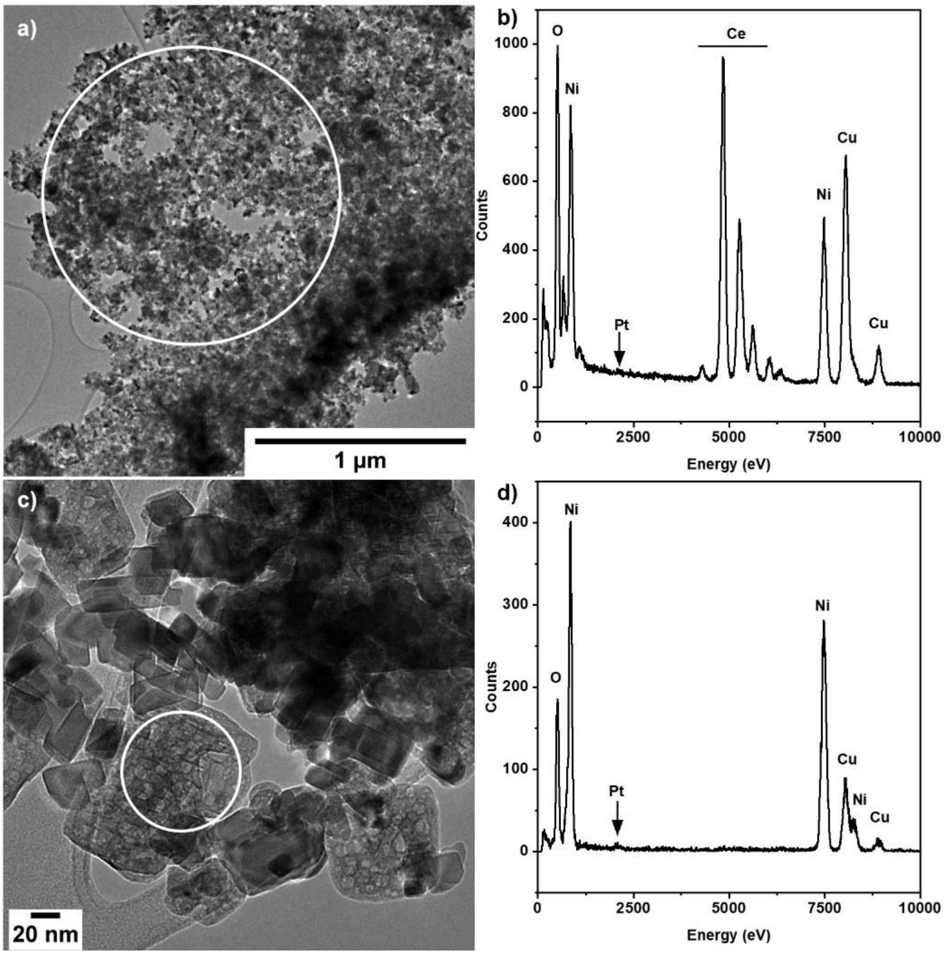

Figure S4. TEM and EDX spectra of a-b) 1-PtNi/CeO_2(--+); c-d) PtNi/CeO_2 seq-IWI

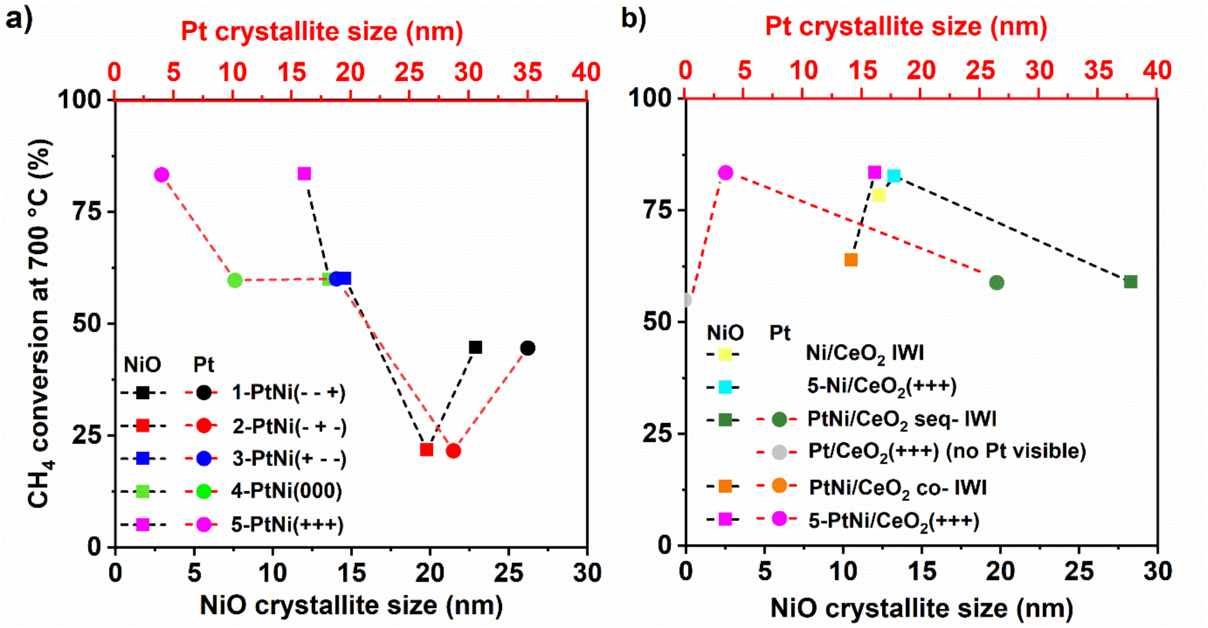

Figure S5. Methane conversion at 700 °C as a function of the NiO and Pt crystallite size estimated by XRD. a) Samples prepared by milling following the design of experiment. b) Samples prepared by IWI and the best milled samples. Monometallic samples are also included. Squares and circles are the data points for the NiO and Pt particle sizes, respectively. Conversion data acquired at a steam-to-carbon = 2, F/W = 202.500 mL gcat-1 h-1.

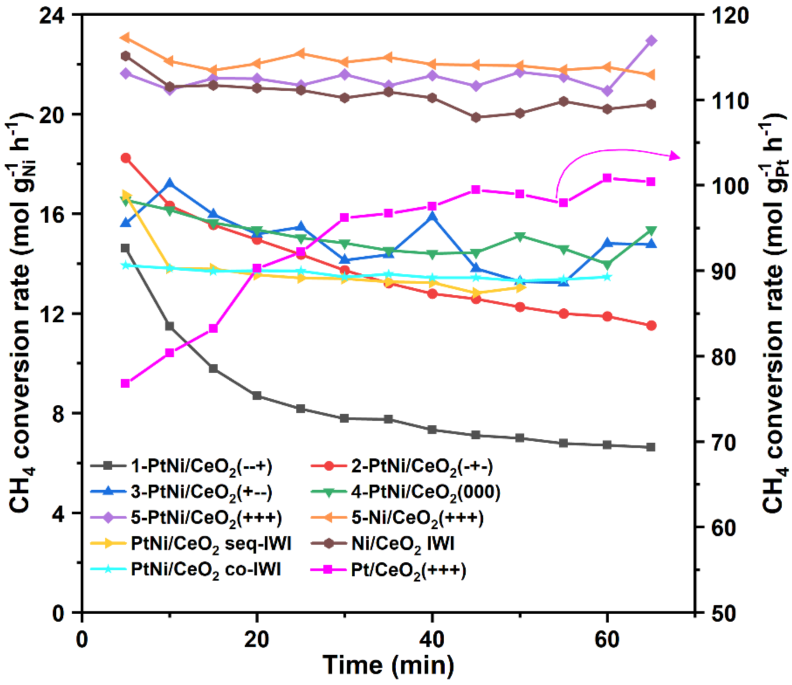

Figure S6. Methane conversion rates during the first hour of reaction at 700 °C for the catalysts tested in this work. Steam-to-carbon = 2, atmospheric pressure, F/W = 202.500 mL g_{cat}^{-1} h^{-1}.

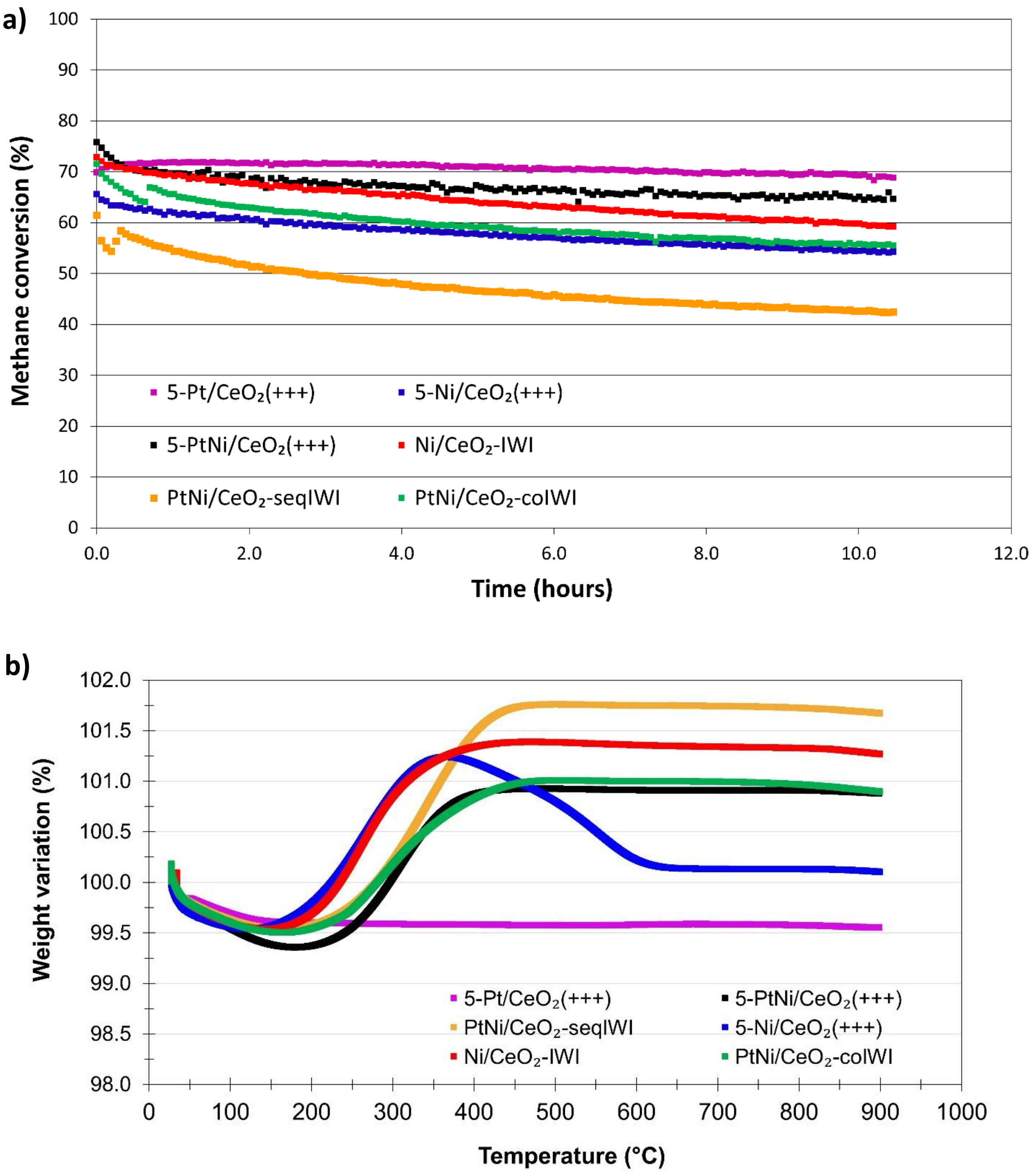

Figure S7. a) Methane conversion as a function of time at 700 °C. Steam-to-carbon = 2, F/W = 202.500 mL g_{cat}^{-1} h^{-1}. b) Thermogravimetric analyses of the samples after the stability tests.

Design of Experiment analysis

In Table S5, the response values are reported together with the DoE synthesis parameters. The data were analysed using Minitab 19 [8] and equations 9 and 10 where obtained from the analysis. These equations represent the mathematical models describing the effect of the three factors (milling frequency, time, and BPR) on the responses. The higher the coefficient associated with a factor, the higher is the effect of that factor to the response value.

Table S5. Values for the two responses (NiO NP size and methane conversion at 700 °C) for the samples prepared with the fractional factorial design of experiment.

Sample name	Frequency (Hz)	Time (min)	BPR	NiO size (nm)	X_{CH4}@700 °C
1-PtNi/CeO_2(--+)	15	5	20	19.8	21.8
2-PtNi/CeO_2(-+-)	15	45	5	22.9	44.8
3-PtNi/CeO_2(+--)	50	5	5	14.6	60.2
4-PtNi/CeO_2(000)	32.5	25	12.5	13.6	59.9
5-PtNi/CeO_2(+++)	50	45	20	12.0	83.5

$$NiO\ NP\ size\ (nm) = 26.27 - 0.23 \cdot Frequency(Hz) + 0.0062 \cdot time(min) - 0.19 \cdot BPR\ (R^2 = 86.8\%)\ (9)$$

$$X_{CH_4}@700\ °C = 3.65 + 1.10 \cdot Frequency(Hz) + 0.58 \cdot time(min) + 0.010 \cdot BPR\ (R^2 = 97.9\%)\ (10)$$

In Figure S8, the main effect plots for the two responses are shown. Each black point is the mean response value at a given factor level from different samples, i.e., the value depicted in the plot for methane conversion as a function of the milling frequency at 15 Hz (33.3%) corresponds to the mean methane conversion obtained for the two samples milled at 15 Hz (21.8% and 44.8%, respectively for 1-PtNi/CeO_2(--+) and 2-PtNi/CeO_2(-+-)). The slope of each plot is related to the coefficients of equations 9 and 10.

Effect of the milling parameters on the NiO nanoparticle size: as seen from equation 9, the factor with the strongest influence on the NiO size is the milling frequency, with a coefficient of -0.23, followed by the BPR with a coefficient of -0.19. The negative signs indicate that by increasing both the milling frequency and BPR values the NiO size decreases. The sample with the smallest NiO size was 5-PtNi/CeO_2(+++), with a milling frequency of 50 Hz and BPR = 20. Both samples milled at the lowest frequency (15 Hz) showed the largest NiO size and the samples milled at 50 Hz showed the smallest NiO size on average. The effect of BPR was less pronounced: considering the variation range for the BPR value equal to 20-5 = 15, the total effect on the NiO particle size was -0.19*15 = -2.85. For the milling frequency, the variation range was 50-15 = 35 Hz, and the total effect was -0.23*35 = -8.05, thus about 3 times higher than the BPR effect. The milling time showed a flat trend suggesting that its effect is negligible, although the central point of the design (4-PtNi/CeO_2(000)) suggests that there can be a curvature in the model with an optimal value of time or BPR that lies in the middle of the investigated parameters. Complex interactions between the parameters should not be excluded, although they cannot be modelled in this study. In addition, the dispersion of the values around the mean is lower for the frequency compared to both milling time and BPR, indicating that the effect of the milling frequency is the most impactful.

Effect of the milling parameters on the MSR activity: similar conclusions can be drawn for the catalytic activity of the catalysts studied. From equation 10, the two parameters with the largest coefficients are the milling frequency and the milling time, with a value of 1.10 and 0.58 respectively. Considering the variation range, the total effect of varying the milling frequency was 1.10*35 = 38.5, while for the milling time it was 0.58*40 = 23.2. Increasing the milling frequency is directly related to an increase of both the number of impacts and the kinetic energy exchanged at each impact,

while the time influences the total number of impacts. The sample with the highest methane conversion was 5-PtNi/CeO_2(+++), which is also the sample with the smallest NiO NP size. [6] The BPR coefficient is about 100 smaller than the milling frequency, suggesting that there is no effect on the catalytic activity, although the main effect plot in Fig. S8b shows that there can be again a curvature in the model. The catalytic properties of 4-PtNi/CeO_2(000) were comparable with those of 3-PtNi/CeO_2(+--) despite being the first milled at lower frequency, probably because of longer milling time and a higher BPR allowed for a similar energy transfer between the ball and the powder (about 3 times higher, see Table 1).

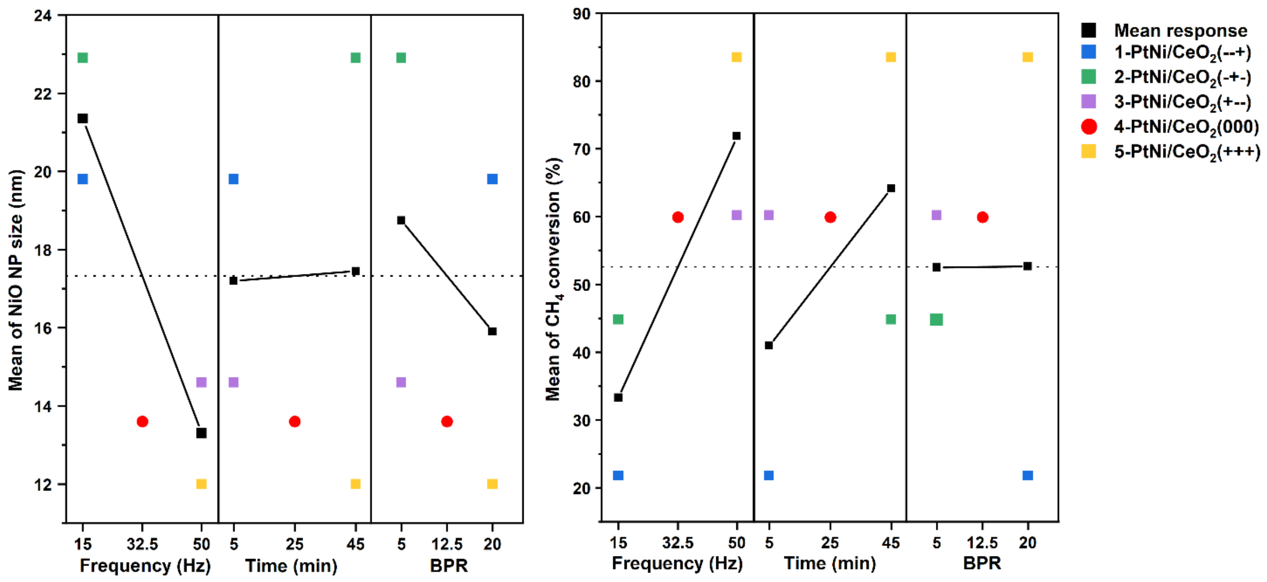

Figure S8. Main effects plots for a) the NiO NPs size and b) methane conversion at 700 °C (Steam-to-carbon = 2, F/W = 202.500 mL g_{cat}^{-1} h^{-1}).

Raman spectroscopy analysis

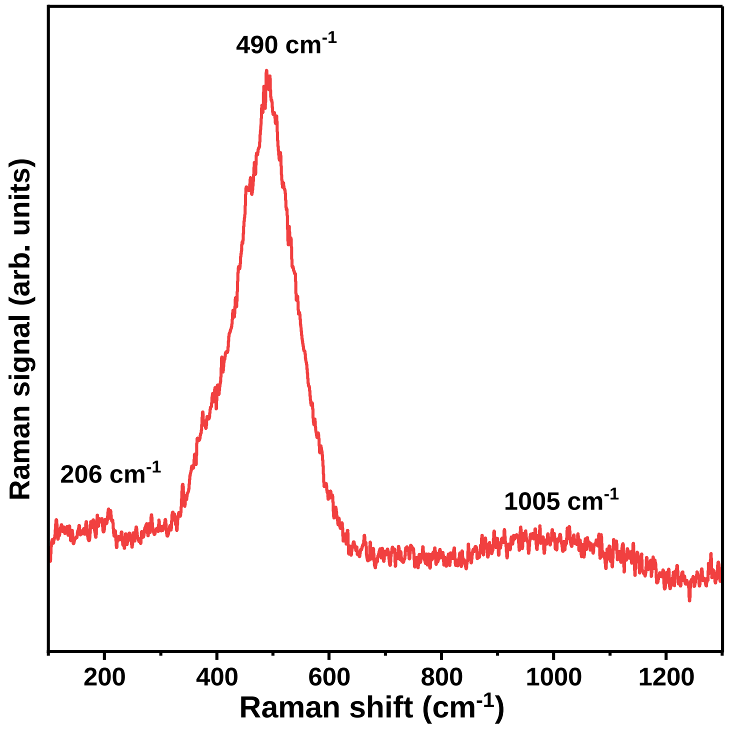

Figure S9. Raman spectrum of NiO prepared by $Ni(Ac)_2$ decomposition at 500 °C. [9] Wavelength = 532 nm, 1 mW, 50x.

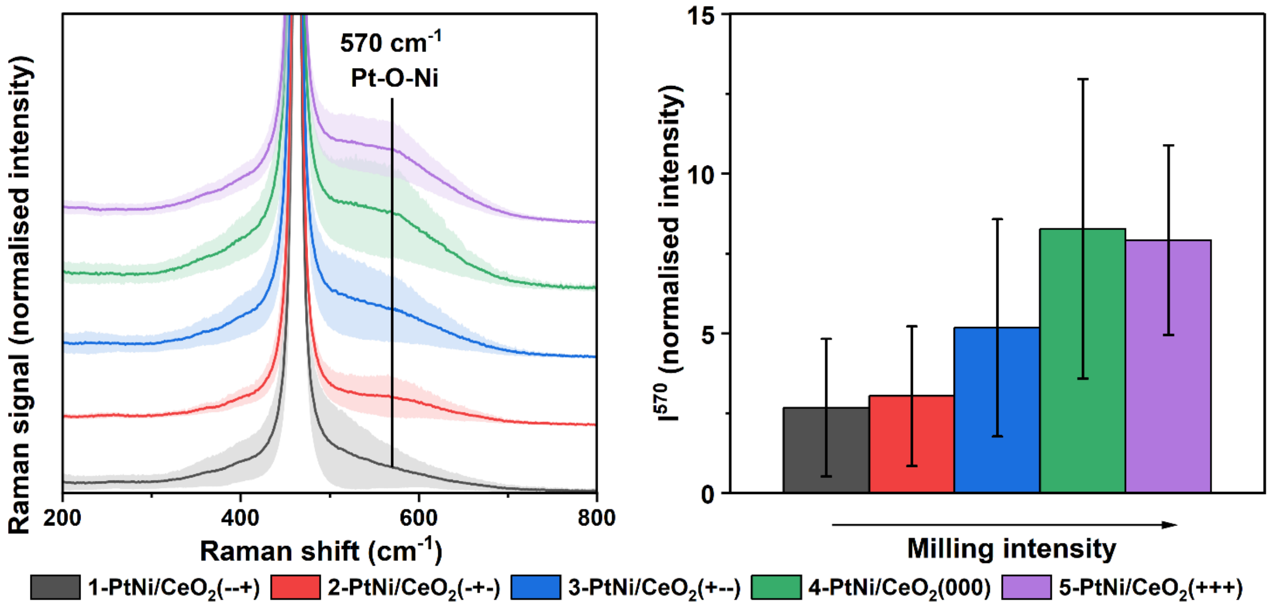

Figure S10. Raman spectra of the DoE milled catalysts, normalised at the CeO_2 F_{2g} peak. The band at 500-600 cm^{-1} associated with the NiO-CeO_2 interaction, and the band at 570 cm^{-1} associated with the Pt-NiO interaction both increased with increasing milling energy. The Raman signals are average values from 7-11 spectra, and the bands correspond to the standard deviation. Wavelength = 532 nm, power = 1 mW, objective = 50x.

XAFS analysis

Table S6. Results of the EXAFS fits. For the fits, the k range was 2.5-9 Å^{-1} and the R range 1.0-2.7 Å for all samples and conditions.

Catalyst	Conditions	Scattering path	CN	$\sigma^2 \cdot 10^3$ (Å^2)	R (Å)	ΔE_0 (eV)	R-factor (%)	Red χ^2
**5-Ni/CeO_2**	AfterTPR	Ni-Ni	10.7±0.6	4.5±0.5	2.47±0.01	-3.4±0.5	0.1	76.7
	AfterMSR		10±1	4.4±0.9	2.48±0.01	-3.1±1	0.2	98.7
**5-PtNi/CeO_2**	AfterTPR	Ni-Ni	9.5±1.4	4.2±1.2	2.48±0.01	-3.7±1.4	0.5	29.8
	AfterMSR		10±1	4.3±0.9	2.48±0.01	-3.5±1	0.3	37.4
**PtNi/CeO_2 seq-IWI**	AfterTPR	Ni-Ni	11.3±0.8	4.8±0.7	2.48±0.01	-3.4±0.7	0.1	25.9
	AfterMSR		10.6±0.7	4.7±0.6	2.48±0.01	-3.2±0.7	0.1	47.4

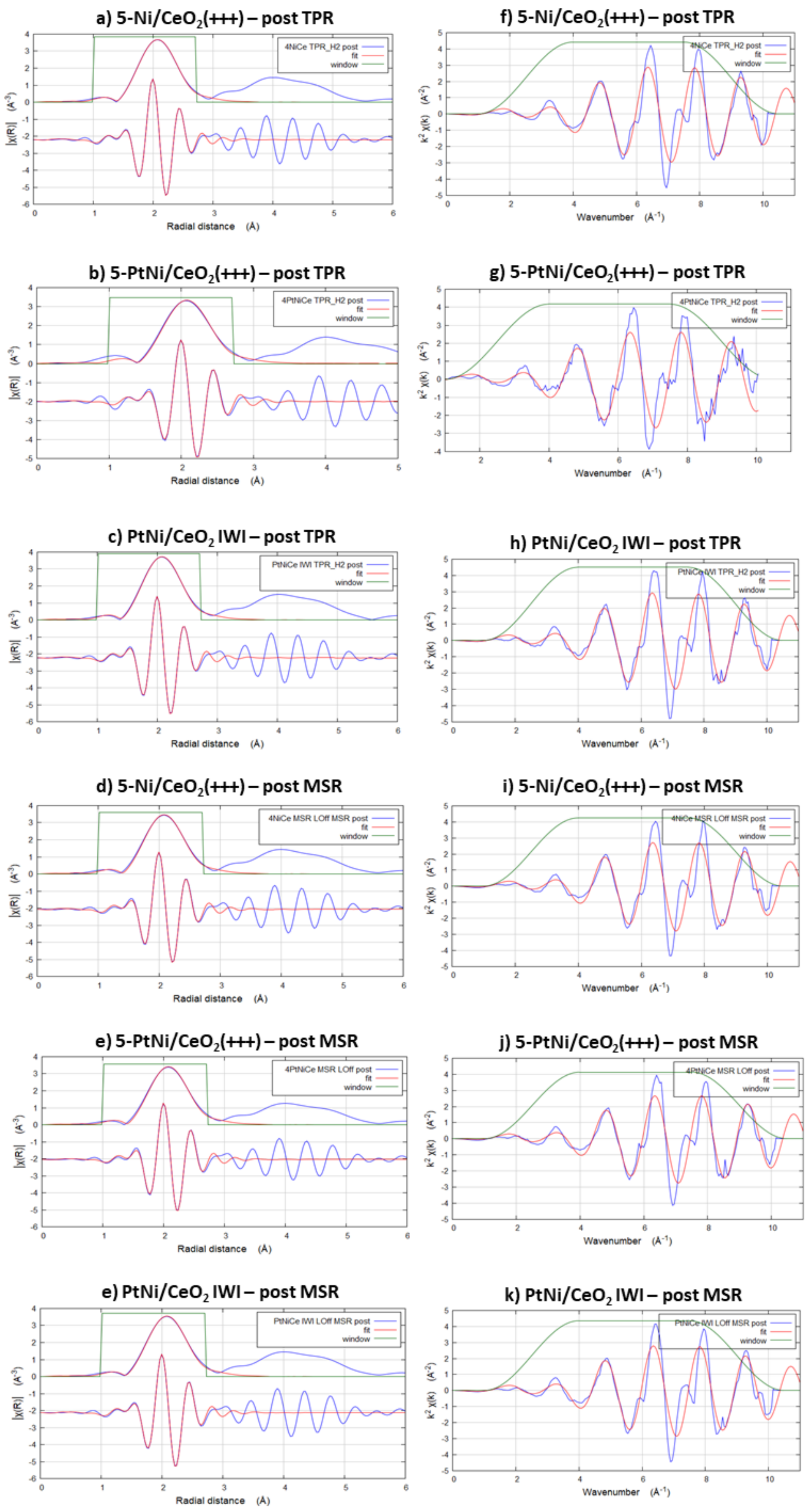

Figure S11. EXAFS fits of the indicated catalysts. a) to e) show the fit in R space and the real part of the Fourier Transform. f) to k) Fit in k^2. Data are k^2-weighted, $k = 2.5\text{-}9$ $Å^{-1}$, not corrected for phase shift.).

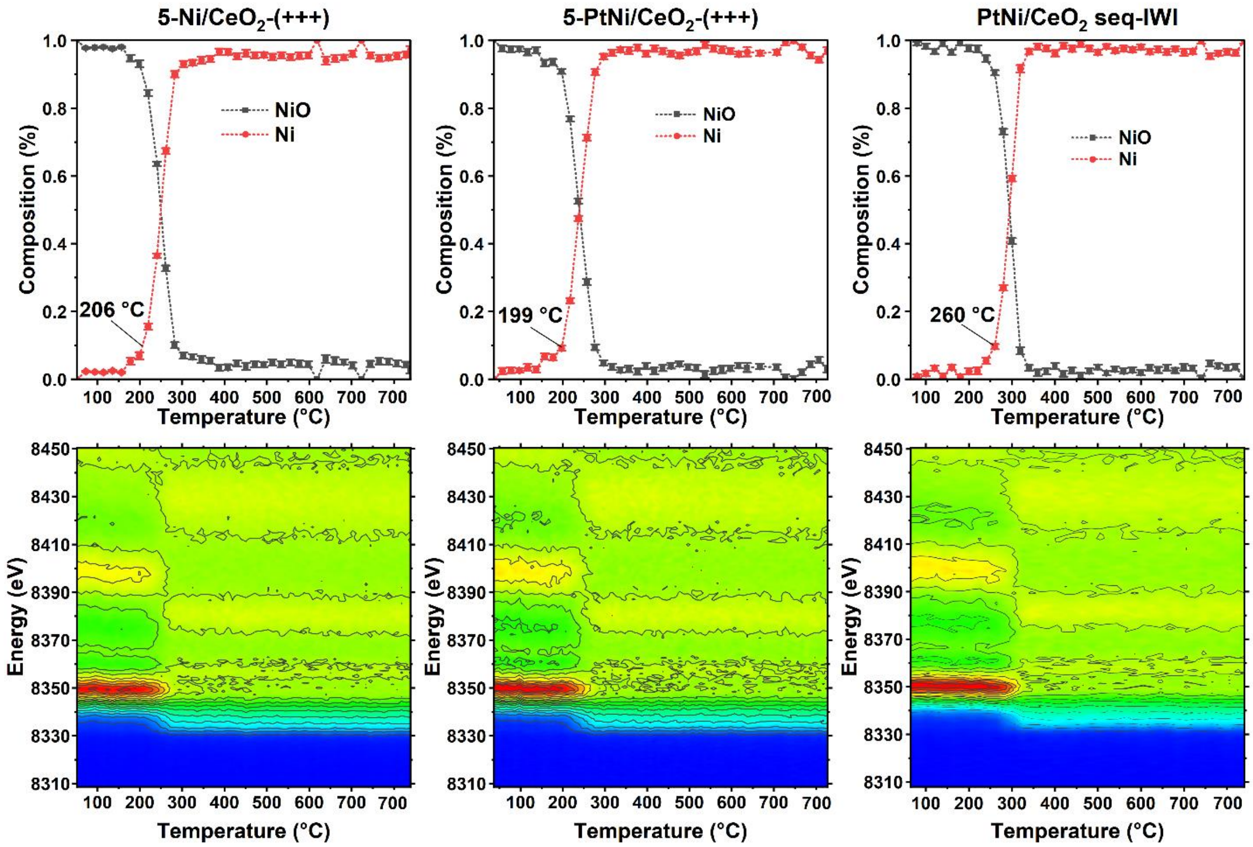

Figure S12. Linear combination analysis of the *in situ* reduction up to 700 °C of the XAS spectra and corresponding 2D XANES maps at the Ni K-edge

NAP-XPS analysis

Table S7. Surface composition of 5-Ni/CeO_2(+++) and 5-PtNi/CeO_2(+++) under the atmospheres indicated measured by NAP-XPS.

**5-Ni/CeO_2(+++)**	**Ce^{3+} at.%**	**%Ce^{4+} at.%**	**Ni/Ce**
O_2 400 °C, 450 eV	12.8%	87.2%	1.27
O_2 400 °C, 215 eV	12.3%	87.7%	1.51
H_2 700 °C, 450 eV	38.2%	61.8%	0.45
H_2 700 °C, 215 eV	54.3%	45.7%	0.52
MSR 700 °C, 450 eV	33.3%	66.7%	0.45
MSR 700 °C, 215 eV	46.9%	53.1%	0.52

**5-PtNi/CeO_2(+++)**	**Ce^{3+} at.%**	**%Ce^{4+} at.%**	**Ni/Ce**	**Pt/Ce**	**(Pt+Ni)/Ce**	**Pt/Ni**
O_2 400 °C, 450 eV	15.9%	84.1%	1.15	0.021	1.17	0.018
O_2 400 °C, 215 eV	15.3%	84.7%	1.42	0.020	1.44	0.014
H_2 700 °C, 450 eV	79.3%	20.7%	0.28	0.004	0.29	0.014
H_2 700 °C, 215 eV	90.4%	9.6%	0.25	0.002	0.25	0.010
MSR 700 °C, 450 eV	72.1%	27.9%	0.30	0.013	0.31	0.044
MSR 700 °C, 215 eV	80.5%	19.5%	0.28	0.009	0.29	0.031

**5-PtNi/CeO_2(+++)**	Ce tot %	Ni %	Pt%
O_2 400 °C, 450 eV	46.2	52.9	0.9
O_2 400 °C, 215 eV	41.1	58.1	0.8
H_2 700 °C, 450 eV	77.6	22.0	0.3
H_2 700 °C, 215 eV	79.7	20.1	0.2
MSR 700 °C, 450 eV	76.1	22.9	1.0
MSR 700 °C, 215 eV	77.7	21.7	0.7

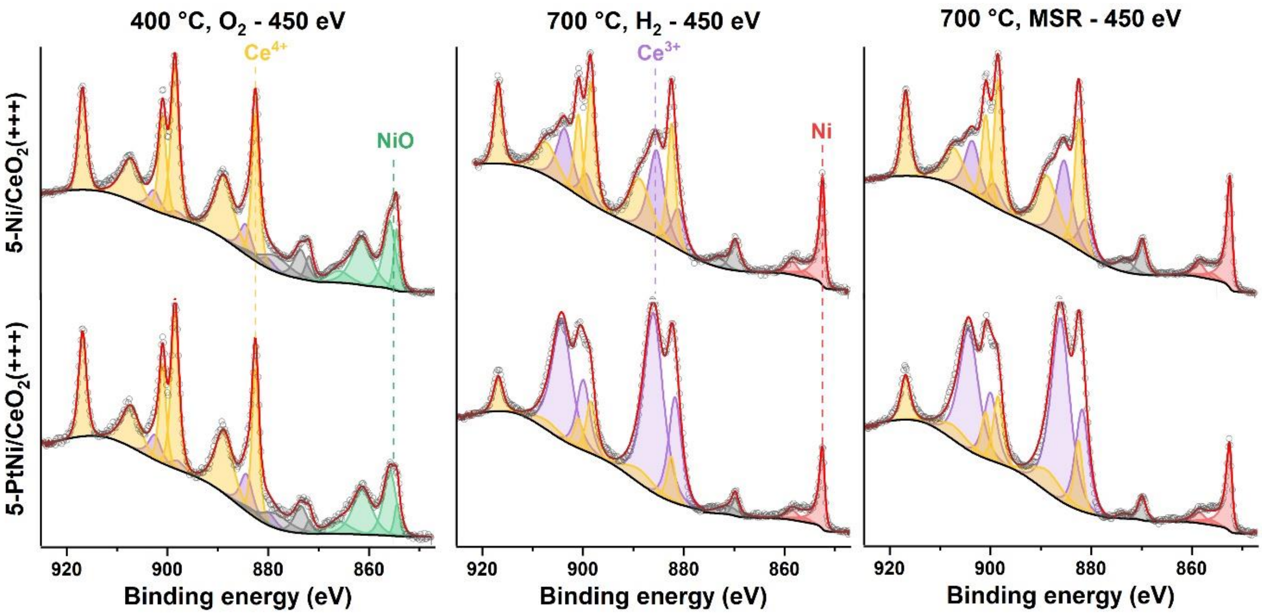

Figure S13. NAP-XPS spectra of the Ce 3d and Ni 2p region of catalysts 5-Ni/CeO_2(+++) and 5-PtNi/CeO_2(+++) measured at a kinetic energy of 450 eV under 1 mbar of different atmospheres. The spectra are normalised.

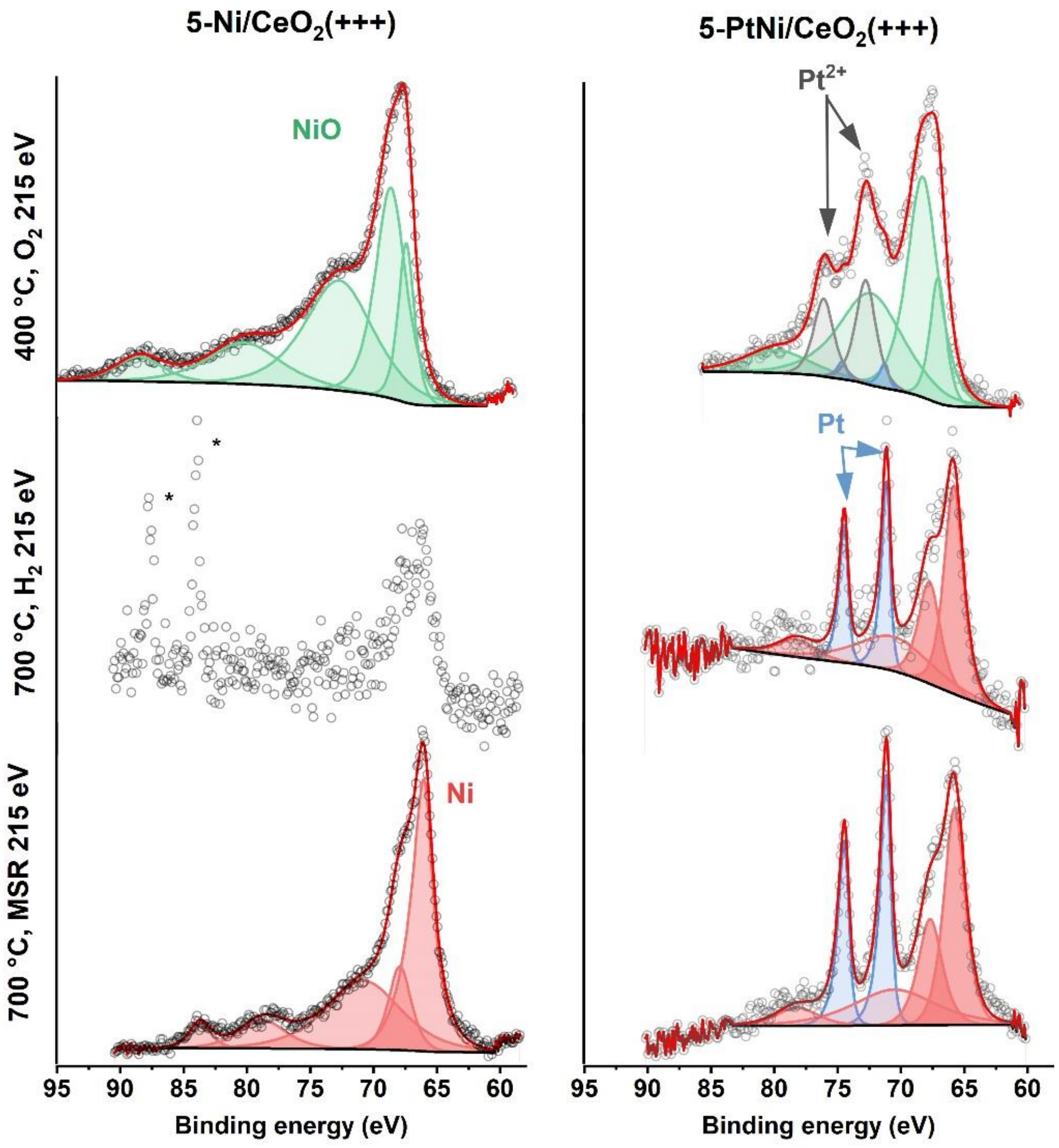

Figure S14. NAP-XPS spectra of the Ni 3p and Pt 4f region of the samples 5-Ni/CeO_2(+++) and 5-PtNi/CeO_2(+++) measured at a kinetic energy of 215 eV under 1 mbar of different atmospheres. The spectra are normalised. The peaks marked with * are Au 4f peaks coming from the Au grid used to prepare the pellets.

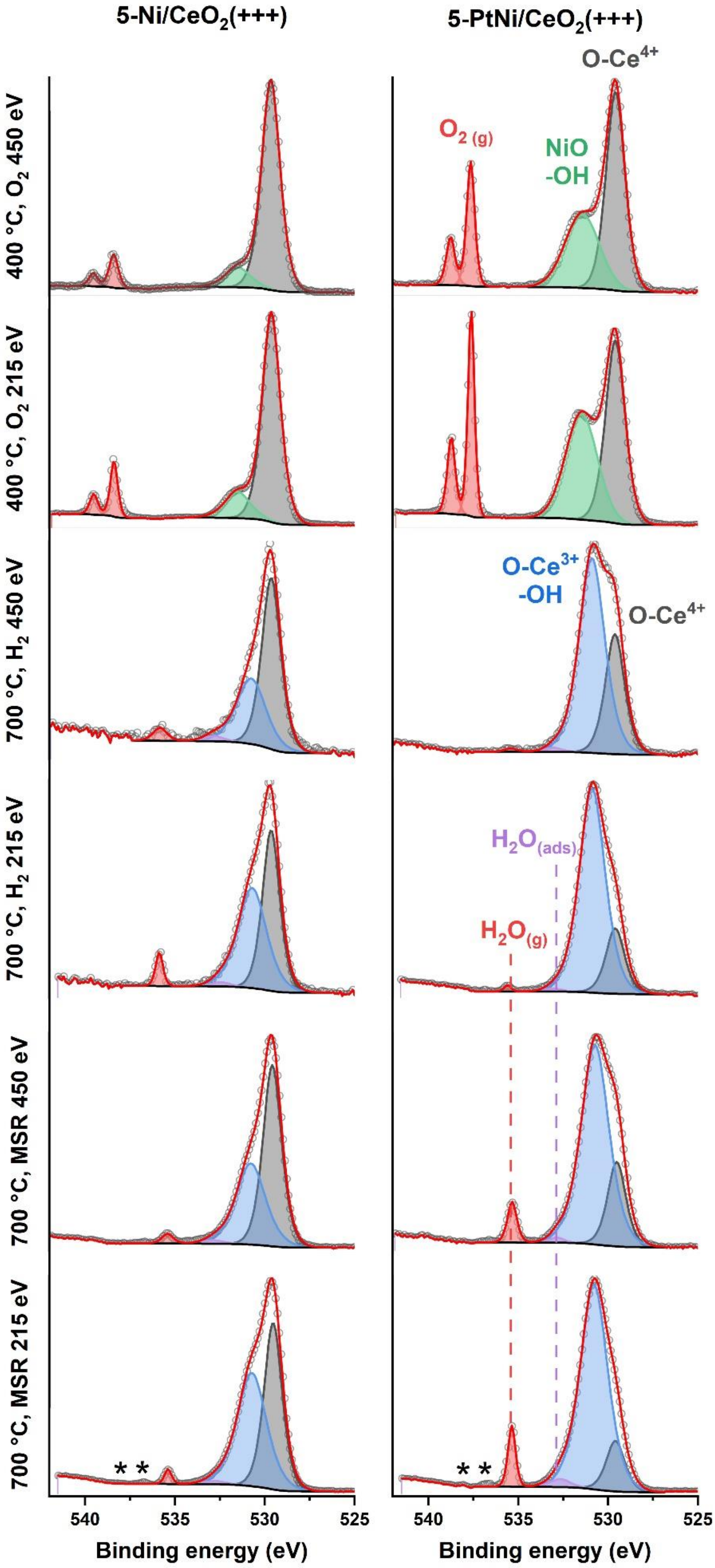

Figure S15. NAP-XPS spectra of O 1s region for 5-Ni/CeO_2(+++) and 5-PtNi/CeO_2(+++) under different atmospheres at 1 mbar of pressure. The position of gaseous CO and CO_2 related peaks are marked with *. The spectra are normalised.

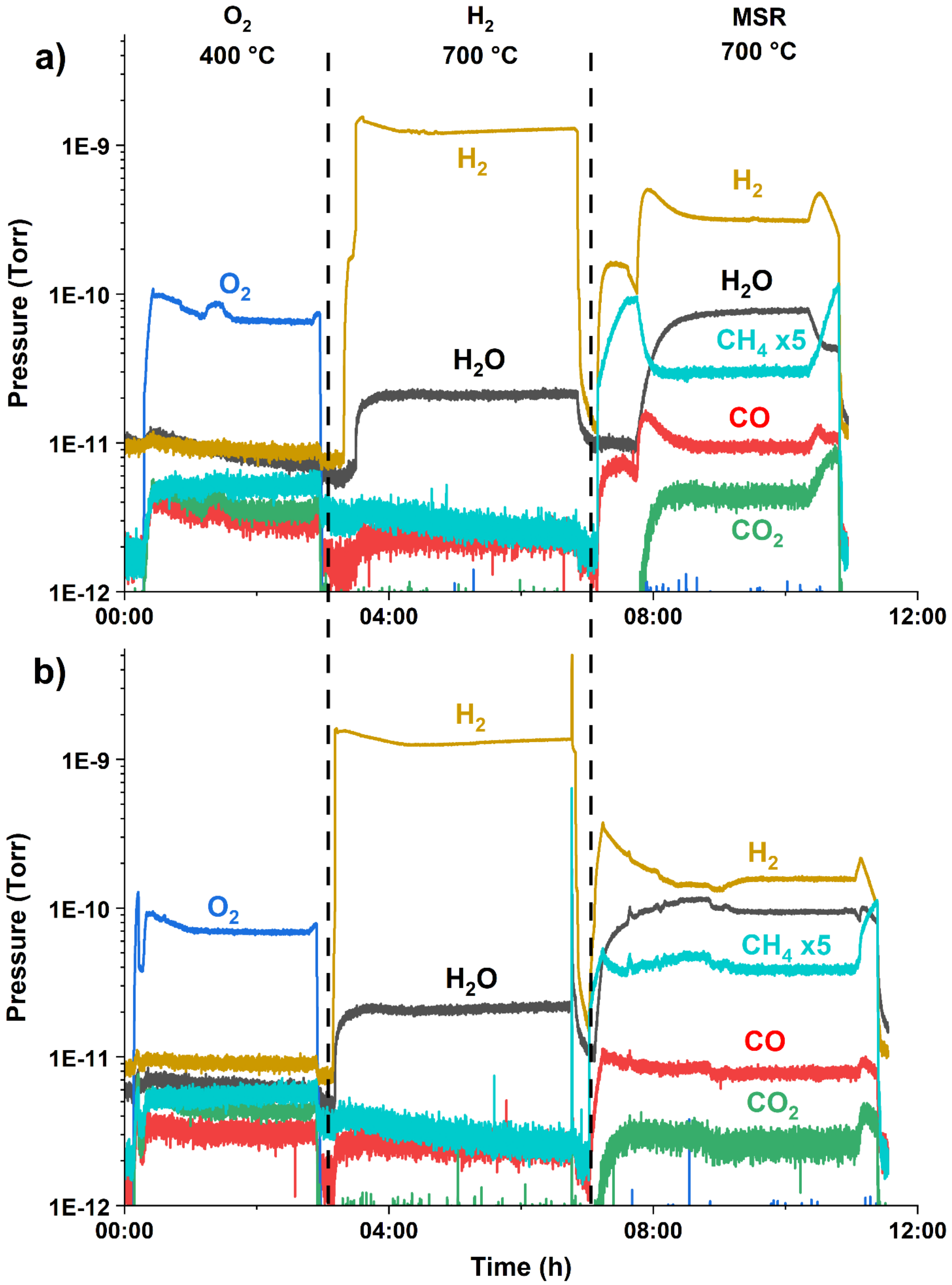

Figure S16. Mass spectrometer signals measured during the NAP-XPS experiments on a) 5-Ni/CeO$_2$(+++) and b) 5-PtNi/CeO$_2$(+++).

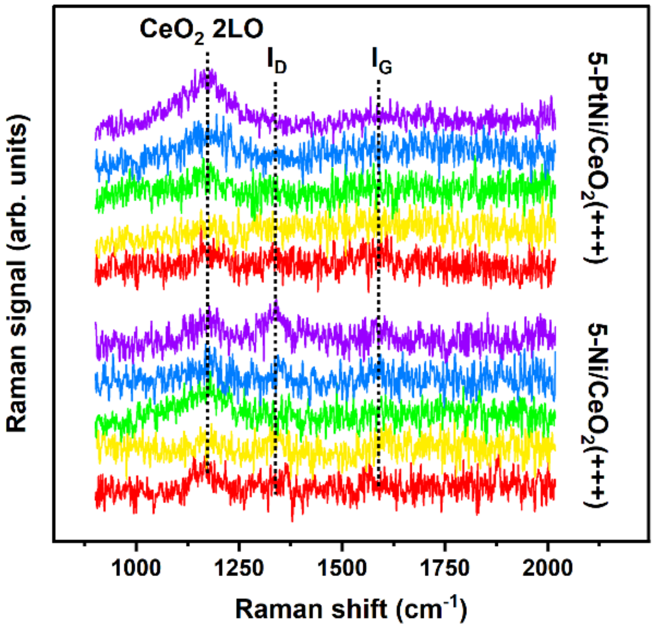

Figure S17. Raman signals of the C region of the spent 5-PtNi/CeO_2(+++) and 5-Ni/CeO_2(+++) catalysts, showing the total absence of C signals from the bimetallic catalyst and weak signals from the monometallic one. Wavelength = 532 nm, 1 mW, 50x, the spectra are normalised with the standard normal variate method. Spectra acquired in different regions of the sample are shown. S/C = 2, T = 700 °C, F/W = 202.500 mL/g_{cat} h

References

[1] C. J. Powell and A. Jablonski, *NIST Electron Inelastic-Mean-Free-Path Database - Version 1.2*. National Institute of Standards and Technology, Gaithersburg, MD, 2010. [Online]. Available: https://www.nist.gov/srd/nist-standard-reference-database-71

[2] D. R. Maurice and T. H. Courtney, “The physics of mechanical alloying: A first report,” *Metall. Trans. A*, vol. 21, no. 1, pp. 289–303, 1990, doi: 10.1007/BF02782409.

[3] M. Magini, A. Iasonna, and F. Padella, “Ball milling: An experimental support to the energy transfer evaluated by the collision model,” *Scr. Mater.*, vol. 34, no. 1, pp. 13–19, 1996, doi: 10.1016/1359-6462(95)00465-3.

[4] G. K. Williamson and W. H. Hall, “X-ray line broadening from filed aluminium and wolfram,” *Acta Metall.*, vol. 1, no. 1, pp. 22–31, 1953, doi: 10.1016/0001-6160(53)90006-6.

[5] R. G. Brereton, *Applied chemometrics for scientists.* John Wiley & Sons, 2007.

[6] C. Vogt, J. Kranenborg, M. Monai, and B. M. Weckhuysen, “Structure Sensitivity in Steam and Dry Methane Reforming over Nickel: Activity and Carbon Formation,” *ACS Catal.*, vol. 10, no. 2, pp. 1428–1438, 2020, doi: 10.1021/acscatal.9b04193.

[7] K. O. Christensen, D. Chen, R. Lødeng, and A. Holmen, “Effect of supports and Ni crystal size on carbon formation and sintering during steam methane reforming,” *Appl. Catal. A Gen.*, vol. 314, no. 1, pp. 9–22, 2006, doi: 10.1016/j.apcata.2006.07.028.

[8] “Minitab, LLC.” 2023. [Online]. Available: https://www.minitab.com

[9] N. Dharmaraj, P. Prabu, S. Nagarajan, C. H. Kim, J. H. Park, and H. Y. Kim, “Synthesis of nickel oxide nanoparticles using nickel acetate and poly(vinyl acetate) precursor,” *Mater. Sci. Eng. B Solid-State Mater. Adv. Technol.*, vol. 128, no. 1–3, pp. 111–114, 2006, doi: 10.1016/j.mseb.2005.11.021.
